# Evidence and evidence gaps in tinnitus therapy

**DOI:** 10.3205/cto000131

**Published:** 2016-12-15

**Authors:** Gerhard Hesse

**Affiliations:** 1 Tinnitus-Klinik, Bad Arolsen, Germany; 2 University of Witten-Herdecke, Germany

**Keywords:** tinnitus therapy, chronic tinnitus, psychosomatic comorbidity, evidence-based medicine

## Abstract

A nearly endless number of procedures has been tried and in particular sold for the treatment of tinnitus, unfortunately they have not been evaluated appropriately in an evidence-based way. A causal therapy, omitting the tinnitus still does not exist, actually it cannot exist because of the various mechanisms of its origin. However or perhaps because of that, medical interventions appear and reappear like fashion trends that can never be proven by stable and reliable treatment success. This contribution will discuss and acknowledge all current therapeutic procedures and the existing or non-existing evidence will be assessed. Beside external evidence, the term of evidence also encompasses the internal evidence, i.e. the experience of the treating physician and the patient’s needs shall be included.

While there is no evidence for nearly all direct procedures that intend modulating or stimulating either the cochlea or specific cervical regions such as the auditory cortex, there are therapeutic procedures that are acknowledged in clinical practice and have achieved at least a certain degree of evidence and generate measurable effect sizes. Those are in particular habituation therapy and psychotherapeutic measures, especially if they are combined with concrete measures for improved audio perception (hearing aids, CI, hearing therapies).

## 1 Introduction

Tinnitus (from the Latin word “tinnire” = ring) is synonymous for ear noise or ear ringing and corresponds to the perception of an acoustic phenomenon that is not caused by external impulses. It is generated at some point of the auditory system, mostly in the cochlea, then it is processed and perceived as noise annoyance in the cortex. Such as all recurrent continuous stimulations, it is habituated in most of the cases by sensory perception, i.e. filtered already subcortically. If this is not possible or if particular attention is paid to the tinnitus, it may sometimes lead to annoyance and also subsequent complaints causing separate disease. Epidemiologic studies for Europe and the United States of America, expect about ¼ of all people having experienced tinnitus sensation at least once, while 10–15% hear tinnitus for a longer interval, actually only 3–5% are considered as requiring treatment, and half of them suffer significantly [1].

In rare cases, tinnitus can be objectified, it is then described as a pulsating noise or a clicking or smacking and corresponds rather to perceived sound produced by the body such as vascular processes or muscle contractions. Much more frequently, subjective tinnitus is observed that cannot be assessed by external measurement and that is caused in more than 90% by a dysfunction of the hair cells in the inner ear and processed as annoying sound [2]. According to recent studies [3], about 15% of all Chinese suffer from tinnitus while the prevalence in higher ages is increased. The main risk factors are hearing loss, middle ear affection, and noise exposure. This leads to significant costs due to the high percentage of patients requiring treatment, mostly for the patients themselves, but also for the healthcare insurances. According to an evaluation of the disease-related expenses [4], the costs amount to nearly 7 billion Euro only in the Netherlands.

The pure disease-related costs for healthcare services are lower compared to the expenses that are caused by social side effects (inability to work, early retirement etc.). In terms of therapy of tinnitus aurium, it is important if its occurrence is acute or already present for a longer time so that it must be considered as chronic. Whereas in the acute stage it is recommended to initiate high-dose cortisone therapy (with moderate but however existing evidence) in analogy to the treatment of sudden hearing loss, there is no known treatment for chronic tinnitus that might causally stop the ear noise.

### 1.1 Tinnitus as noise in the auditory system

93% of all tinnitus patients have concomitant (doubtfully causing?) measurable hearing loss, 44% complain also of hyperacusis. Most ear noises are observed in the high frequencies and impose as high whistling sound; they nearly always correspond to a simultaneously existing hearing loss in those high frequencies [5], [6].

Even for acutely arising hearing disorders, tinnitus is observed often only in phases of relaxing or after a longer interval. In contrast, tinnitus is suddenly subjectively perceived in the context of slowly developing deafness that occurs in the majority of the cases, often triggered by stress or emotional distress. Because of this close relationship with the hearing perception, tinnitus aurium is not considered as an independent disease but as a symptom of disturbed hearing at any location of the auricular system.

Based on the cortical network, especially the combination with emotional aspects and negative assessment, annoying ear noise develops and as a consequence also comorbidities such as concentration and sleep disorders, partly even depressive episodes and anxiety. Those comorbidities are actually significant for the development of an independent disease; they lead to the fact that the tinnitus aurium must be considered as complex disease, especially in cases of patients requiring treatment. In terms of therapy, this has a significant impact because only mechanistic, symptom-related approaches have little prospect of success due to this complexity.

### 1.2 Tinnitus and hearing loss

In the majority of the cases, tinnitus is associated with hearing loss. Sometimes this hearing loss is subjectively not perceived at all or as annoying effect especially when it has slowly developed. According to own data, tinnitus patients only rarely have regular hearing capacities [7]. This means that tinnitus is mostly observed in the frequency of the highest hearing loss, mostly imposing as high-frequency sound because of the dominance of high-frequency hearing impairment. Sudden hearing impairment as for example in the context of sudden hearing loss or noise trauma is often accompanied by tinnitus that often occurs only in the interval, i.e. when the hearing impairment improves or remains on a certain level.

Thus, tinnitus corresponds to a primary functional loss of mainly external hair cells of the inner ear as described recently by Noreña [8] in a review article. However, mostly enhancement mechanisms in the auricular system are responsible for the perception of tinnitus that enhance this frequency via cortical reactions and priming or reduction of lateral inhibition or increase of base frequencies so that the impression is dominant. This means that a peripheral dysfunction leads to central tinnitus enhancement or tinnitus accentuation. In accordance, often the distortion products of otoacoustic emissions are nearly paradoxically increased [9], [10].

In terms of diagnostics, it is necessary to identify exactly possible hearing impairment even if the patient himself considers his hearing capacities as regular. Beside the pure tone audiogram, in particular the assessment of the function of the external hair cells is reasonable and required by measuring DPOAE.

Only very rarely, when the hearing capacity is completely regular, also after measuring OAEs, the ear noise will have to be understood as a consequence of general over-stimulation and thus incorrect processing of the central auricular system.

### 1.3 Studies on tinnitus therapy

In summary, the trial situation of scientific evaluations of therapeutic approaches and results is unsatisfactory. Four main circumstances are responsible for this fact:

1. Since there is no clear origin for ear noise and tinnitus is rather a symptom and epiphenomenon of an impaired hearing perception, there is consequently no clear patho-physiological explanation. This means that also therapeutic approaches cannot be uniform.
2. According to current knowledge, pharmacotherapy or surgery in the sense of “switching off” tinnitus are not possible. Of course, accompanying or even triggering diseases can be treated but a causal therapy removing the phenomenon of tinnitus does not exist and will probably never exist in the near future.
3. At the same time, the treatment of tinnitus represents an important market as confirmed recently by studies conducted in the USA [11], [12]. So numerous treatment providers are present on the “tinnitus market” that beside serious, scientifically proven approaches offers also various unserious, even paramedical treatment concepts.
4. Actually, tinnitus only requires treatment when comorbidities occur that are mostly classified as psychosomatic such as sleep disorders, concentration disorders, but also anxiety and depressive episodes. Often those side effects lead to the urgent need of treatment even if the symptom of tinnitus is always considered as triggering factor and the patient expects palliation for exactly this symptom.

All this means that tinnitus therapies – if they are expected to have positive results – never work as mono-therapies, especially not under time-restricted conditions of regular ENT practices that only rarely allow sufficient information. However, this fact makes it problematic to scientifically evaluate solid and valid trials because the single therapeutic effects cannot be clearly related to the one or other measure. So it is evident that good and successful tinnitus treatment must be combined with compensation of parallel and accompanying hearing loss, in general by prescribing hearing aids. Up to now, the evidence that hearing aids are useful for the treatment of tinnitus could not be proven because this treatment is always only part of the therapy and cannot be assessed separately. Thus, those trials are not considered in meta-analyses.

In reality, many therapies and approaches evaluating single measures scientifically are always successful with combined therapeutic modules such as the training of relaxing techniques or psychological counseling and stabilization, often even psychotherapy in scientifically sound procedures. Then often only one therapeutic element is in the focus, the others are “forgotten” in order to relate therapeutic success to only one treatment. However, from a scientific point of view this is not “neat” and falsifies the data situation.

Generally, studies on the efficacy of pharmaceutics are only rarely placebo-controlled, often one group of substances is compared to another which neglects the placebo effect that is extremely high in the context of tinnitus [13]. The scientific importance is thus clearly reduced. Often pharmaceutical or instrument-related therapies are performed in ENT practices as so-called observational trials – without control and validated evaluation. From the start, those studies are useless, nonetheless they are often taken as basis for commercial advertising. 

Even more problematic are therapies that are based on technical solutions such as for example radiation or acoustic alienation. In this context, often technical inspection certificates are mentioned as quality proof that certify a general possibility to be used in a medical context to a device (especially because it does not cause direct harm), however, no clear statement can be given on the therapeutic success and the actual benefit for the patient. While reliable studies in three clinical phases are required for pharmaceutics, this rule is not applied to technical devices. So nearly every device that does not cause damage can be sold as therapeutic instrument and promoted as such.

From experience, that scientific assessment of such therapies and devices is very difficult, sometimes trials are mentioned in single cases, but also in this context the possibilities of control and especially the placebo-controls are extremely difficult if not even impossible. For example, even for non-experts a placebo coil is recognized as not being active in the context of transcranial magnetic stimulation. The same applies for tonal stimulation that then places stimuli at totally different locations than corresponding to the own tinnitus.

Thus, a placebo effect is seen and as a consequence it is not really present.

Only very few trials meet the requirement that is the base of medical evidence. Actually the question must be asked if trials included in according meta-analyses really meet the criteria without simply omitting certain therapeutic aspects, as described above. Better evidence is found for trials on the treatment of chronic tinnitus that (nearly always) concern cognitive behavioral therapy.

Even if often special conditions are valid for such trials, e.g. because only less stressed patients were included, the significance is not reduced regarding the statement that the evaluated elements from cognitive behavioral therapy are effective in patients suffering from tinnitus. So they are – as published in the manuals by Kröner-Herwig [14] and Delb [15] – useful parts of each serious tinnitus therapy.

However, the meta-analysis of Martinez-Devesa [16] for example and other studies with limited study design must not lead to the claim of superiority of “cognitive behavioral therapy” for outpatient individual therapy or in daily routine [17].

It still remains scientifically unsatisfactory that representatives of psychodynamic therapies have not submitted evaluated investigations on the evidence for tinnitus going beyond case reports [18], [19], [20].

In particular, cognitive behavioral therapies – especially when manualized – can be very well assessed and thus also standardized. For according meta-analyses this is always a very important criterion leading to the fact that other comparably effective (also psychotherapeutic) therapy approaches do not reach the same degree of evidence.

In summary, in the discussion of scientific investigations, the current preconditions for evidence determination are still not a really reliable tool to differentiate effective from non-effective measures. Hence, the “old” requirement remains [21] that probably only networking and central assessment of many different therapeutic approaches and centers may provide reliable data.

### 1.4 Evidence and evidence gaps

The topic of this contribution are evidence and evidence gaps in tinnitus therapy. In the context of medicine (evidence-based medicine), evidence means proof.

During the last years, evidence-based medicine turned out to be an independent science which is the gold standard especially for evaluation of therapies and therapeutic recommendations even if also this aspect is not without controversy. Evidence-based medicine is built on three pillars:

1. The current state of knowledge of clinical medicine is based on clinical trials and medical publications (external evidence).
2. In the clinic, this external evidence must be in accordance with the individual experience of the physician and
3. the needs and requirements of the patient.

According to Coyle [22], the preconditions for external evidence are fulfilled only in well-structured and high-quality studies conducted in a randomized, controlled, double-blind, and generally also placebo-controlled way with sufficiently high numbers of patients. Those investigations are classified according to validity criteria into more or less strong evidence.

With regard to tinnitus therapy, already those preliminary reflections lead to numerous problems:

- Especially pharmaceutical therapies are often applied based on traditional customs and experiences without having sufficiently studied data. Since those therapies are often performed in general or specialized practices, high case numbers are usually not available or not collected.
- Since the patients often perceive a very high individual level of suffering, randomization is rather difficult, in particular in cases of intensive psychotherapeutic or even inpatient treatment.
- This is especially true for placebo-controlled treatment because generally patients refuse undergoing placebo treatment due to the mentioned level of suffering.
- In the context of therapies that consist of talking, personal experience, or basic exercise, placebo control is nearly impossible or would not be ethically justifiable.
- Evidence for a therapy regime in the sense of meta-analyses can only be achieved if single therapeutic interventions could be compared. However, often therapeutic regimes are applied consisting of several arms (such as for example psychotherapies, audiotherapies combined with relaxation techniques for chronic tinnitus) for the treatment of chronic diseases that generally cannot be treated. Thus only rarely or even no isolated data can be collected for the single therapeutic arms that refer only and exclusively to this one therapeutic arm.
- Finally the typical measuring instrument of randomized, controlled studies in the sense of a “surrogate marker” for tinnitus therapy is a special questionnaire and less specific measurements results. Since tinnitus remains mostly unchanged in terms of intensity and also tone pitch and there is little correlation to individual annoyance [23], those individual questionnaires assess the actual stress. The questionnaires are subjective in the true sense of the word and correspond to individual, subject-related assessment. They are validated [24] but are still based on the individual assessment of the patient. Clinical assessment and questionnaires in the sense of evaluation questionnaires by physicians or therapists do not exist. Thus these tools can only be used in a limited way for measuring the success of trials even if they are currently considered as the international gold standard for measuring therapy success. Especially in English-speaking countries, frequently visual analog scales are applied that are even more subject-related and suggestive.

Nowadays, the bases for evidence-based medicine are so-called meta-analyses representing a summary of primary investigations that are evaluated in a qualitative and statistical way. For such meta-analyses, different trials in a research field are studied, then summarized, and statistically evaluated. The investigations primarily included should preferably be homogenous in order to allow consistent data collection and then to analyze them in a statistically sufficient way.

For studies on tinnitus therapy, especially those homogenous research objects are rarely found because in particular chronic tinnitus is influenced and characterized by numerous factors that are relevant for the actual appearance and suffering from the symptom. So, the research objects are rather heterogenic which is a main criticism according to Feinstein (cited after Weßling [25]) in the sense that such heterogenic characteristics cannot be summarized satisfactorily in a meta-analysis. Furthermore, trials with negative therapeutic effects are generally less frequently published (publication bias). In 2008, Turner et al. [26] could well show this aspect based on an investigation of the efficacy of antidepressant medication. They elaborated that numerous registered studies have not been published while it is not clear if they had not been accepted for publication or not submitted at all. Subsequent meta-analyses thus often have false positive assessments. This fact mainly concern studies and meta-analyses that are sponsored by pharmaceutical companies [27]. But if – such as often in the context of trials on tinnitus therapy – only little proof and comparable studies exist, clear evidence and good reasoning is nearly not possible: criteria of evidence-based medicine are not fulfilled although some therapeutic approaches might be effective. According practice guidelines that only refer to the basis of evidence-based medicine thus cannot recommend those therapeutic approaches. This fact is well elaborated in a publication by Kern et al. [28] (describing the example of physical medicine and general rehabilitation): The authors explain that the original definition of evidence-based medicine included individual clinical experiences beside external evidence, however, that this gets increasingly in the background and only external evidence is considered as the most important guideline for financial compensation of therapies and therapeutic guidelines.

The authors refer in particular to pain therapy that can certainly be compared to the therapy of chronic tinnitus and criticize that often clinically proven therapies are doubted or not approved. This becomes obvious regarding the fact that according to El Dib et al. [29] 96% of a total of 1024 review articles do not give a definite statement on existing or missing evidence.

Finally it is important to mention that the conduction of large, multicenter, and high-quality studies causes significant costs. Since in general pharmaceutical companies are not available as sponsors (because success cannot be expected) for tinnitus therapy studies and state funding is rather reluctant, those are other reasons explaining why there are only few reliable trials in this context.

Nonetheless, an extremely high number of studies is published even if only very little data is present which furthermore is not even reliable. In many countries, research and even part of the clinical work is financed via funding, hereby publications are essential to have access to funding. Additionally, numerous online platforms facilitate publication. Potential authors are “invited” to submit studies, rapid processing is promised. The authors have to co-finance these kinds of publications: often, however, a peer-reviewing does not work soundly. Furthermore, the high number of submitted articles worldwide causes a certain tiredness among the reviewers. If someone has to check the data of a trial thoroughly, this needs time and learning the matter. So it can be expected that also the reviewers only marginally verify many studies. This aspect is obvious regarding the fact that even some high-quality journals (with high impact factors) often accept trials that are methodically false or show significant flaws and do not even meet the basic criteria of scientific working and ethical obligations. This also means that the basic rule of anonymous submission is often neglected – certain authors may then publish nearly everything, regardless of actual quality and scientific and especially clinical relevance. Due to the internet, today nearly all studies are available on a worldwide scale, most of them at least via the abstracts. However, those abstracts are often too short and especially in the context of therapy studies they sometimes describe conclusions that actually cannot be drawn from the trial and are not even mentioned explicitly. Since many authors only read the abstracts for their discussion chapters, systematic errors are included and distributed. 

Sometimes, in particular in the context of publications from China and Korea, only the abstract is available in English, the trial itself is published for example in Chinese language. Of course, this explains why so many studies are not considered for meta-analyses.

If this situation that lasts already for a long time really leads to evidence gaps or if this gap can be partly closed by clinical expertise, remains to be observed and finally also to be discussed in the last part of this chapter.

## 2 Classification of tinnitus disease and stress

### 2.1 Preliminary remarks

Patients claim from themselves and even more from their treating physicians to give understandable causal explanations about the symptom of tinnitus. Ear noises may occur in various forms, as pure sounds of different frequencies, as sound mixtures or as narrow band or broadband noise. The quality of the ear noise is highly important for the patient, however, it is not really relevant for the pathological correlations. Generally it must be said that most of the ear noises impose as highly frequent whistling. This fact also allows conclusions on the accompanying or triggering high-frequency hearing loss. In contrast, low-frequency buzzing sounds are often associated with low-frequency hearing loss and may be a hint to an endolymphatic obstruction in the inner ear. Furthermore, the tinnitus may be intermittent or permanent, its intensity may vary, at least regarding the individual perception, and may even by pulsating in single cases. For the systematic history taking it is also important to know if the ear noise can be enhanced by movements of the head, neck, or jaw or if physical movement reduces or increases the tinnitus.

Beside the already mentioned differentiation between objective and subjective tinnitus and tinnitus with or without hearing loss, it is important for therapy if tinnitus occurred acutely or if it is observed already for a longer time and especially if it is compensated or decompensated, which means if and to what extent already comorbidities have developed.

### 2.2 Acute – chronic

Especially with regard to therapeutic interventions, the duration and persistence of the ear noise are highly relevant. An acute tinnitus occurring for the first time often disappears spontaneously after a short time or after according therapy. Only if the ear noise persists for more than 3 months, it is called chronic. This also depends enormously from the attention the patient pays to the phenomenon. The classification of acute and chronic is important because it defines if acute therapies is still successful in this phase of the disease. According to the common guidelines and therapeutic experiences, an acute therapy is only useful within the first 3 months [30], [31].

Hereby it is important that even in the context of chronic tinnitus of longer durations there are situations when tinnitus becomes louder or more intensive. This aggravation which is commonly called exacerbation, however, cannot be compared to acutely occurring tinnitus. It is rather only a perception phenomenon that is triggered by certain situations such as stress.

Thus it does not make sense to start acute therapy with cortisone infusions or even hyperbaric oxygenation during those so-called exacerbations of a chronic tinnitus.

### 2.3 Compensated – decompensated – comorbidities

Finally, it is decisive for therapy and the need of therapy of the tinnitus patient how he is able to cope with the phenomenon and to what extent he suffers. If we expect that more than 25% of all Germans have already experienced tinnitus but only 2% feel impaired by the ear noises, there are numerous patients who rapidly accept their tinnitus as given and habituate. The ear noise does not cause an urgent need of therapy and is not perceived as annoying. Therefore they do not hear it permanently. Regular habituation processes in the hearing processing compensate the tinnitus, completely independent from the timely phase or the duration of its presence. However, if the impression of tinnitus is combined and enhanced in the brain by plastic alterations and linking in the emotional assessment and consecutively by focusing reactions, the regular habituation is impeded and suffering from the tinnitus results [32]. Tinnitus tends to decompensate or already decompensates the patient. The symptom then dominates the affected person, controls his ability to live and decide and impairs it more or less intensively [5], [33]. This possible development is completely independent from the circumstance if the tinnitus was generated primarily in the inner ear, in the hearing nerve, the brain stem, or the central hearing processing.

More or less frequently, this increasing focusing leads to psychosomatic comorbidities such as sleep and concentration disorders. Primarily tinnitus is made responsible for them because the patient can no longer concentrate due to the tinnitus, can no longer sleep due to the tinnitus, and even wakes up due to the tinnitus. Often social isolation and depressive reactions develop and again tinnitus is made responsible for this situation, although certainly also accompanying exhaustion, stress, and a general depression may be the origin.

Another frequently occurring comorbidity is anxiety caused by the fact that tinnitus is seen as threatening symptom and fear of aggravation or new damage develops. Often it is associated with a hypersensitivity to noises (hyperacusis).

### 2.4 Significance of this classification for therapy

In cases of objective or objectified ear noises that are actually very rare, a basic pathophysiology exists that may even be treated surgically. For example pulsating ear noise may be caused by arterio-venous fistulas or vascular processes. In this context, it must be discussed if surgery is appropriate and necessary, which depends on the degree of real tinnitus severity and also of probably resulting comorbidities. 

However, it is important to differentiate between acute and chronic tinnitus because according to the current study situation and to the actualized guidelines pharmacotherapy should only be attempted in the really acute stage whereas it is no longer useful in cases of chronic tinnitus.

For therapeutic reflections, it is crucial to evaluate the actual distress caused by the ear noise and the developing side effects and comorbidities. If an ear noise does not really disturb the patient and if it is not made responsible for other disorders, therapy is not really required. The ear noise will then be suppressed simply by habituation processes. The basic knowledge about the significance of the chronic tinnitus and its assessment as a disease still remains that the actual persistence of tinnitus and consequently the developing distress are only due to cortical plasticity [8], [34]. Even if originally a hearing damage, especially damage of the external hair cells of the inner ear is present, the processing in the auricular system and the interconnection with emotional and evaluation qualities in the auricular system that are basic for the significance and the pathology of this ear noise. In a certain way, also the conflict is resolved hereby that came up during the last years with regard to the genesis of ear noises. Especially we as ENT specialists expect a primarily peripheral genesis in the hair cells of the inner ear while e.g. neurologists and psychiatrists indicate preferably the central significance and erroneously state that tinnitus is centrally generated. It is correct in this context that distress caused by tinnitus develops and is generated centrally, however, the ear noise itself develops at different locations and mostly really in the inner ear. This is important for therapeutic options that have to take into consideration the primary genesis as well as the further central processing.

### 2.5 General reflections on tinnitus therapy studies

In general, the methods of studies on the efficacy of cognitive and neuro-otologic psychosomatic therapies improve continuously while trials on the direct influence of the tinnitus – either by pharmaceutics or by cortical modulations – are often extremely superficial and methodologically rather poor. Then too readily successes are announced that have to be withdrawn shortly afterwards or the according therapies have already disappeared from the market. However, this situation has been observed for tinnitus therapy for more than 40 years. An interesting review article from the USA was published in 2013 [35]. Via internet, more than 9000 trials and publications between 1970 and 2012 were assessed, but only 52 could be evaluated in terms of therapeutic effects in cases of tinnitus. Among those publications, 17 evaluated pharmacological therapies, 11 dealt with other interventions such as TMS or laser treatment, 5 with sound therapy, and 19 with approaches of psychological behavioral therapy. The authors complain about the aspect that nearly no data is given on side effects. In total, they found only weak evidence for the efficacy of cognitive behavioral therapy with regards to tinnitus-related improvement of the quality of life. Weak evidence was further seen regarding the loudness of the tinnitus for the efficacy of neurotransmitters compared to placebo. Insufficient evidence was found for the efficacy of antidepressants, other pharmaceutics, and food supplements regarding loudness of the tinnitus and all other therapeutic goals. Insufficient evidence was also identified for acoustic neuro-stimulation, rTMS, and sound therapies, partly the studies had a high bias, for example caused by direct economic involvement of the authors in selling the evaluated medical devices. But this review article also mixed up possible therapeutic effects and did not differentiate if the topic was the distress caused by the tinnitus (which can be assessed by specific questionnaires) or direct audiological factors such as loudness and frequency of the tinnitus. A meta-analysis from Nottingham submitted in 2011 [36] considered 28 randomized and controlled studies on tinnitus therapy and addressed crucial subjects: many of those trials had poor evidence because they had not been blinded, their significance was not well measured, and additionally the data was often not completely reported. Also in this context, those were only trials on cognitive behavioral therapy that were sufficiently large and thus comparable and that could assign a certain – even if modest – effect size to this type of therapy. In this evaluation, a certain evidence for antidepressants was found, however, it was not clear of what the therapeutic effect of antidepressant for tinnitus really consisted. 

Also in 2011, a review article on tinnitus therapy was presented in a neurologic journal that could not have been more superficial [37]: For therapy of chronic tinnitus numerous reasonable but also inappropriate, non-confirmed therapies without scientific assessment were presented. According to this review article, nearly everything seems to be effective, starting with hearing aids, noisers, and cochlear implants, but also pharmaceutics such as gingko and caroverine, low-power laser therapy up to cognitive behavioral therapy. If therapies are compared in such a confuse way and therapeutic success is reported even in review articles without any comment, it does not help finding useful therapeutic decisions. In many review articles and meta-analyses the criticism is found that there is none of the common treatment approaches for chronic tinnitus working as monotherapy, generally multimodal approaches are applied and then evaluated. However, the scientific assessment for the single therapeutic options becomes difficult and even impossible. This is also true for the often emphasized cognitive behavioral therapy because this treatment is nearly always combined with other procedures as for example hearing therapies or relaxation exercises. In general, those are not included in the evaluation and quasi concealed. Additionally, blinding or even placebo therapy for those therapeutic approaches is impossible. In 2012, a multidisciplinary study group tried to make a methodological proposal for improved studies [38]. Even this proposal of an international standard for tinnitus therapy correctly states some of the problems, but it does not really consider the main problems of scientific evaluation of tinnitus therapies:

1. Many therapies cannot be performed in a placebo-controlled or double-blinded way because they are effective cognitive or psychotherapeutic approaches. The attempt to try less effective or reduced procedures (as placebo or control) does not correspond to controlled therapy studies. So-called sham therapies such as radiation, magnetic or electric stimulation are immediately recognized as placebo by the patients.
2. As already explained, nearly all therapies are conceived as multimodal treatment, i.e. complementary therapeutic moments are added to the examined issue. Those are for example counseling, information, or also sound diagnostics with intensive information of the patient. The main difficulty consists of differentiating them methodologically or excluding them. However, in the trials they are mostly not assessed.
3. The real effectiveness is identified only after long-term effects, short-term improvement such as 15–20% reduction of the intensity are de facto irrelevant and generally do not persist. The cited proposal for consensus [38] contains only the comment that longer-lasting trials would be difficult to conduct. Of course this is true but especially catamnesis studies are the ones that are really missing regarding tinnitus therapy.

In parallel, there are especially the patients who – if bothered by tinnitus – require preferably causal therapy stopping the tinnitus. Also in this regard there are interesting studies that focus on the actual readiness of the patients to undergo certain therapeutic options. Rich Tyler from Iowa dealt with the questions what patients would be ready to tolerate to make the tinnitus more bearable [12]. According to this trial, at least 19% of the examined 197 patients would accept an implant in the brain if they completely lost their tinnitus. 13% would even accept such a treatment if the tinnitus was half as loud. The readiness to take pills amounted to more than 50% for both questions. Most patient would spend up to 5,000 $ to lose their tinnitus, 20.3% would even spend more than 25,000 $.

In a subsequent trial, 439 patients were asked again: 40% had already spent between 500 $ and 10,000 $. 70% would accept implantation of a device or stimulator to just reduce the distress caused by tinnitus [11].

Those preliminary reflections shall only give an impression how tense the field of tinnitus therapies is and which expectations patients as well as therapists often have regarding short-term relief. However, evaluating more intensively the multiple possibilities of the origin of ear noises, especially the development of real and clinically assessable distress for the patient, it is clear that mechanistic proposals that focus only on one aspect have to be directly excluded. But this does not happen because such mechanistic approaches are pursued due to different interests. Frequently the results are methodologically “cleaned up”. Sometimes even secondary or tertiary effects are first assessed in so-called post-hoc analyses that identify an effect even if primarily no effect can be confirmed as for example in subgroups of hypertension patients or patients suffering from tinnitus caused by middle ear disease.

Hence, even numerous trials often cannot confirm an evidence of the according therapy. But if classic evidence in the sense of external evidence is not necessarily required for effective tinnitus therapy, many of the presented studies of more clinically oriented assessment of the efficacy lead to no or even negative significance.

In this article, single therapeutic approaches for acute and chronic tinnitus will be presented and valued, with regard to possible or also missing evidence and also with regard to a relevant therapeutic significance for the ENT specialist.

### 2.6 Practice guidelines

Since 2014, there is a “Clinical Practice Guideline: Tinnitus” [39]; it contains definitions and a total of 13 therapy recommendations. An “executive summary” [40] explains the methods of the recommendations and suggests evaluation studies and further evaluations.

The recommendations are:

- Targeted history taking and physical examination
- Intensive prompt audiologic examination
- Differentiation and assessment of the real distress caused by tinnitus
- Information and education about management strategies
- Hearing aid evaluation if useful
- Recommendation of cognitive behavioral therapy in cases of bothersome tinnitus
- Recommendation against antidepressant, anticonvulsants, anxiolytics, or intratympanic medications
- Recommendation against ginkgo, melatonin, zinc, or other dietary supplements
- Recommendation against transcranial magnetic stimulation (TMS)
- Optional: audiologic examination and subsequent recommendation to hearing or sound therapy

In this US-American guideline, some therapies are explicitly marked as “recommendation against” because there are no reliable study data. Therapies for which the evidence of existing trials is not sufficient, are described at least as “optional” as for example hearing and sound therapy.

In contrast, the German S3 Guideline: Tinnitus from 2015 [31] reports about confirmed evidence of tinnitus therapies. According to this document, only tinnitus-related counseling and cognitive behavioral therapy can be recommended as evidence-based treatment. However, this guideline generally recommends cognitive behavioral therapy and not only “manualized tinnitus-related cognitive behavioral therapy”, as Zenner [41] erroneously writes in his summary for the journal HNO.

Also in this context, pharmacotherapy is not recommended, neither alternative therapies or oxygen therapies or magnetic radiations; the recommendation to hearing aids, cochlear implantation and hearing or sound therapies remains open according to this guideline because there is no sufficient evidence. Recommended as evidence-based is the treatment of comorbidities, especially anxiety and depression.

Practice guidelines may be very helpful for treatment especially when – as in the USA – therapies are classified directly as not being useful. In Germany, there is no such negative valuation (partly because of fear of prosecution) and only therapies are recommended in the guideline for which a certain evidence is confirmed. Of course this also means that other approaches, in particular the many and partly very expensive instrument-based procedures, cannot be recommended.

## 3 Pharmacotherapy of tinnitus

Until a few years ago, medication stimulating the blood flow was considered as standard of tinnitus treatment – not only in the acute stage. Agents such as pentoxifylline even have (until now) an approval for the treatment of hearing disorders and sudden hearing loss; a high-dose ginkgo extract (Tebonin^®^ 120 mg for ear noises) is approved for adjuvant therapy of tinnitus of vascular and involutive origin [42] – whatever this may be. For both agents, however, there are no scientific proofs for efficacy, nonetheless both substances are still frequently prescribed in cases of chronic tinnitus but not paid by the statutory health insurances. At the same time, numerous other drugs are tested and compared with one another in trials, rarely with placebo; often – mostly sponsored by pharmaceutical companies – recommendations are given that do not withstand scientific evaluation or even meta-analysis.

### 3.1 Pharmaceutics in the acute stage

#### 3.1.1 Therapy of acute tinnitus

In accordance with the guideline [31], the therapy of acute tinnitus follows the treatment of acute sudden hearing loss. The same problems are present in both diseases: evidence-based therapy regimes are rarely found, actually the generally concomitant acute hearing loss is treated (with high-dose cortisone, systemic or intratympanic application). If tinnitus occurs acutely without hearing loss, the standard therapy is not recommended because psychosomatic factors such as overstimulation certainly play a decisive role [43]. Even more recent therapeutic approaches such as the intratympanic treatment have no effect on tinnitus as described in a study from Istanbul [44]:

70 adult patients with acute tinnitus underwent randomly either intratympanic methylprednisolone or saline solution injection. The treatment was applied once per week for three weeks. Both groups had no pretherapeutic differences regarding age, sex, or tinnitus severity and loudness. After treatment, a significant reduction of tinnitus loudness was observed in both groups. The tinnitus severity, however, did not change significantly. In both groups, pains were observed as side effect, additionally a burning sensation and bitter taste were seen in the prednisolone group. The authors could not confirm a therapeutic benefit, unfortunately, no statement was found on the origin of possibly concomitant hearing loss.

In another study from Korea, 139 patients with acute tinnitus were treated with intratympanic injections of dexamethasone on 4 consecutive days. The patients had no noise-induced hearing loss and no clear (>30 dB) acutely occurring hearing loss. 43 patients (37.7%) lost their tinnitus, 42 had improved results, and in 29 patients the tinnitus remained constant. The authors concluded a good prognosis after intratympanic dexamethasone (ITD) injection [45], however, no placebo-control was performed so that the significance of this study is low.

#### 3.1.2 Intratympanic application of AM-101 in acute tinnitus

Regarding the treatment of acute hearing reduction, a trial was published on intratympanic therapy with the transcription activator bound to hyaluronic acid AM-111, a significant success could not be confirmed [46].

The basis for similar pilot studies are research protocols according to which NMDA receptors are up-regulated in stress situations of the inner ear and cause increased activity of the neural fibers, possibly also tinnitus. In a pilot study [47], conducted in a multicenter, double-blind, randomized, and placebo-controlled way, the NMDA antagonist esketamine hydrochloride (AM-101) was injected intratympanically in different dosages in 24 patients with acute tinnitus persisting not longer than 3 months. The tinnitus severity was measured by means of the tinnitus questionnaire TQ-12 and assessed 60 days after therapy for the last time. None of the dosages and neither the placebo could improve the tinnitus severity. The self-rated tinnitus loudness and the MML (minimal masking level) changed moderately, also in the placebo group. The authors consider this therapy as a good and safe therapeutic option with tendency to improvement of the tinnitus. The fact that the tinnitus questionnaire as only evaluated measuring instrument did not reveal any improvement, is explained by the aspect that some patients had bilateral tinnitus but only one side was treated so that the distress persisted.

Consequently, esketamine hydrochloride (AM-101) was further evaluated in larger multicenter trial for treatment of acute tinnitus [48]. This study was double-blind, prospective, and placebo-controlled, 248 patients between 16 and 65 years were treated with 3 intratympanic injections of AM-101 (high- or low-dose) or placebo on 3 consecutive days. No significant improvement was observed, only patients with tinnitus after noise-induced hearing loss and after middle ear infection experienced improvement with the drug compared to placebo. During the follow-up time of 90 days, the subjectively perceived tinnitus loudness was better with AM-101.

In another study performed afterwards in 2015 on this NMDA antagonist, the best dosage was found [49]: in 16 centers in the USA and Europe, 85 patients who had tinnitus for not longer than 3 months after noise-induced hearing loss, barotrauma, acute middle ear infection, or middle ear surgery, underwent intratympanic injection of the drug or placebo in the context of a phase II study conducted in a double-blind, randomized, and placebo-controlled way. Half of the patients received one treatment, the others had 3 injections in weekly intervals (placebo or AM-101). In summary, a low effect size was measured regarding the improvement of tinnitus loudness, more clearly for the group with 3 injections. The follow-up time was 90 days. Regarding the subjectively perceived tinnitus severity measured in an analog scale and the severity measured by means of the tinnitus questionnaire, the effect was much lower. Undesired side effects were mainly local reactions, however, in 17% the tinnitus even deteriorated and 12% had a hearing reduction that occurred due to the intervention and was regressive afterwards. The authors conclude that this treatment is appropriate, probably and in contrast to the previous study as acute treatment also for other tinnitus origins.

Both trials are methodologically well performed, but they both do not consider sufficiently the hearing loss and its improvement under therapy. With intratympanic AM-101 treatment, the hearing threshold improved significantly but this fact was not correlated with the improvement of the tinnitus. The efficacy of this NMDA antagonist regarding acute tinnitus is not clear because it is not very convincing if primarily no significant differences are observed, but in a post-hoc analysis groups are found where an effect can be confirmed and then a general efficacy is concluded. In general, tinnitus after middle ear surgery or inflammation does not frequently occur, rather after noise-induced trauma. But in this context, cortisone therapy is also effective. According to this study with intratympanic application of the NMDA antagonist, there was no improvement for the patients because the tinnitus severity did not change, only its loudness improved. This parameter, however, is completely individual and also scientifically only a difficult measure that does not correlate with tinnitus severity. It is astonishing that many authors tend to include a positive recommendation in their abstracts even if actually no effect of the therapy could be confirmed.

Another rather deterrent evaluation regarding pharmacotherapy of acute tinnitus after sudden hearing loss is presented by a Korean group:

107 patients with sudden hearing loss and tinnitus were randomly assigned to 3 groups: group 1 received only alprazolam, a benzodiazepine with middle duration of action. Group 2 also received alprazolam and 4 intratympanic cortisone injections, and group 3 additionally received 4 injections of lipo-prostaglandin E. Group 2 (benzodiazepine + intratympanic injection) had the best results, it improved of 75%. In 25.8% of the cases, the tinnitus completely disappeared, the same was observed in 20% of group 3, but only in 9.8% of group 1 [50].

The authors of this article do not explain why all patients received benzodiazepine that is considered as causing depression itself beside a high potential of addiction. Such a therapy regime cannot be recommended; even the trial is useless with regard to such a drug cocktail. The fact that it could actually be published (after all it was even *The Laryngoscope*) does not stand for thorough work of the reviewers!

### 3.2 Pharmaceutics for chronic tinnitus

The above-mentioned statements on acute tinnitus are all the more true for pharmaceutical treatment attempts of chronic tinnitus – currently there are no serious options of pharmacotherapy. Although the legislator requires far-reaching and valid studies for the approval of pharmaceutics for certain indications not only in Germany, a high number of drugs are evaluated with regard to their efficacy in chronic tinnitus – however those are nearly exclusively comparative studies. Hereby several or two drugs are compared in terms of their efficacy, only rarely placebo-control is performed. Especially studies that are promoted by pharmaceutical companies are reluctant regarding a comparison to placebo. This aspect was obvious in studies conducted during the last years on the efficacy of the massively promoted ginkgo extract. 

In the following paragraphs, single studies will be in the focus while the few placebo-controlled studies will be reported at the beginning. Afterward evaluations on different substances and substance groups will be presented.

#### 3.2.1 Placebo-controlled studies

Piribedil, a dopamine agonist, does not significantly improve tinnitus. In a double-blind, placebo-controlled, prospective cross-over study conducted by a team from Regensburg [51] randomized 100 patients with chronic tinnitus to undergo therapy with 50 mg of piribedil and placebo for 90 days each. Piribedil is a dopamine agonist and is applied in Parkinson’s disease. 56 patients finalized the study, the therapeutic success was assessed via the tinnitus handicap inventory (THI) and visual analog scales. In comparison to placebo, no significant improvement could be achieved with piribedil therapy. However, the electro-cochleographic findings and also DPOAE were conspicuous in the piribedil group and different from those of the placebo group: some patients developed a double peak in the electro-cochleography in the CAP, they also had better responses to the therapy, which it was not significant. A suppression of the DPOAE observed in some patients could not be further correlated.

#### 3.2.2 Vardenafil, a PDE5 inhibitor has no effect on chronic tinnitus superior to placebo

A high-quality pilot study was conducted at the Charité in Berlin as a double-blind, prospective, randomized, and placebo-controlled trial on the efficacy of a PDE5 inhibitor in tinnitus patients [52]. 42 patients with chronic tinnitus received either 10 mg of vardenafil twice per day for 12 weeks or placebo tablets. The therapeutic success was assessed by means of the tinnitus questionnaire. Neither the questionnaire total score nor the single subgroups showed a significant improvement of the vardenafil group compared to placebo. The authors conclude that the vasodilative effect and the cGMP increase induced by the agent have no impact on the tinnitus symptoms even if hypoxia and oxidative stress play a role in the genesis of tinnitus. Severe side effects were not described, but prolonged erection and swellings of the nasal mucosa were observed. Furthermore, headaches, vertigo, and facial flushing occurred. An influence of the hearing capacity was not observed, neither positive nor negative.

This study should have set an end to the discussion of blood flow in the context of chronic tinnitus, since a clearly higher blood flow of the peripheral vessels can be achieved by vardenfil which is also shown by the side effects. The study is methodologically sound and conducted in a controlled way, however, it is surprising that soft parameters such as effects on sexual life or partnership are not mentioned in the original article. Probably they are responsible for the originally positive statements of the patients that were the reason for the trial.

#### 3.2.3 Anticonvulsants have no positive effect in the treatment of tinnitus

A Cochrane meta-analysis [53] assessed and valuated 7 trials on the treatment of chronic tinnitus with anticonvulsants encompassing a total of 453 patients. The studies evaluated gabapentin, carbamazepine, lamotrigine, and flunarizine. None of the studies showed a significantly positive effect for one of the pharmaceutics. One trial revealed a significantly negative effect of gabapentin. One trial showed a – non-significant – positive effect of carbamazepine compared to placebo, another one showed an – also non-significant – negative effect. The same is true for flunarizine, positive and negative effects were revealed but without significance. Side effects were described for all tested substances in 18% of the patients. This Cochrane meta-analysis clearly confirms that anticonvulsants are not only ineffective but sometimes even deteriorate the tinnitus-induced distress.

#### 3.2.4 Neramexane – trends for improvement of stress due to tinnitus

In several centers, the tolerability and dosage of neramexane, an NMDA receptor antagonist were evaluated based on a very complex, methodologically sound, randomized, double-blind, and placebo-controlled study [54]. A total of 431 patients with chronic tinnitus persisting for 3–18 months were included in this study. The tinnitus severity was assessed with the tinnitus questionnaire TQ12, audiometric data were collected. A dosage of 50 mg as well as 75 mg could achieve a light, however not significant improvement. Significant improvement was only achieved with the dosage of 50 mg 4 weeks after the end of therapy. Thus the neramexane study showed an improvement only at a second glance, 4 weeks after the end of therapy. During treatment, the tinnitus-induced distress did not change. Often an intensive counseling and information about the findings took place in the final conversation, such a counseling itself already has a therapeutic value and might explain modest post-therapeutic improvement. The authors recommended a phase III study, also conceived as multicenter trial, but it was withdrawn probably because the postulated therapeutic success was too vague and not permanent.

#### 3.2.5 Melatonin – improvement of stress and sleep disorder due to tinnitus, especially in male patients

In a prospective, randomized, double-blind, cross-over, and placebo-controlled study from Ohio, USA, 61 patients suffering from tinnitus for more than 6 months were treated with 3 mg melatonin or placebo [55]. The tinnitus severity was assessed by means of the American TSI questionnaire. The mean age was 57.8 years. After melatonin application, 57% of the patients reported improved symptoms, but also 25% of the placebo group. The better effect was achieved in male patients, in cases of bilateral tinnitus, and in patients who had not received previous treatment and who were not depressive. The same percentage of 57% observed improved sleep after melatonin application (36% after placebo). Side effects were not registered. Follow-up did not take place, the last values were assessed 4 weeks after the end of therapy. Completely disappeared tinnitus was not described.

A review article from San Antonio [56] emphasized the positive effect of melatonin especially as protection in the context of aminoglycoside and cisplatin treatment, but also for the treatment of tinnitus.

A review article from Italy analyzed studies evaluating the treatment of tinnitus patients with melatonin [57]. In 5 trials, therapy success could not be found with regard to tinnitus, however, the sleep disorders improved.

At least a weak positive effect without side effects was documented, but the follow-up time of 4 weeks is rather short and complete disappearance of the ear noises did not occur, as in none of the other studies as well.

#### 3.2.6 Antiemetic drugs for ear noise

In a multicenter trial from England [58], a new antiemetic and anxiolytic drug (vestipitant) was tested in 24 adult tinnitus patients. The study was randomized, double-blind, placebo-controlled, and cross-over. It was conducted for 14 days, the tinnitus severity was assessed by means of the VAS and THI. In summary, no improvement under medication was observed, the intensity of the tinnitus even significantly increased.

Furthermore, the antiemetic drug of ondansetron was examined that is expected to be effective against tinnitus and to even improve the hearing threshold, as reported by an ENT team from Teheran [59]. In a randomized, double-blind, and placebo-controlled study, 30 patients with tinnitus persisting for more than 3 months, with and without hearing loss were treated with ondansetron and compared to a placebo group of the same size. The hearing loss was reported as only moderate over all frequencies without documenting concrete results. Regarding the tinnitus, the THI (tinnitus handicap inventory), TSI (tinnitus severity index), and VAS (visual analog scale) were assessed, in addition to the Hospital Anxiety and Depression Index (HADS) and the thresholds. While VAS and THI, anxiety and depression were not significantly different in both groups, the TSI significantly improved after drug therapy. Surprisingly, the hearing and speaking thresholds improved under ondansetron application (4–16 mg/d for 4 weeks), even only of 3 dB on the average. The hearing threshold improved more in high frequency hearing impaired patients, however, it was neither documented nor specified. The authors explain the hearing improvement and the associated improvement of the tinnitus with a blockade of nicotinic acid receptors in the external hair cells and thus an improvement of the cochlear enhancement. Follow-up beyond 4 weeks did not take place. The result that antiemetic drugs may be applied for hearing improvement, is surprising. However, a summarized improvement of the hearing threshold of 3 dB over all frequencies without differentiation and documentation does not really prove an improved inner ear performance. Further it remains totally unclear why only one questionnaire shows changes and none of the others and why a positive effect of the therapy is concluded nonetheless. Again: how could such a trial be accepted by the reviewers for publication in a scientific journal?

#### 3.2.7 Studies of single agents

Intratympanic cortisone therapy for refractory tinnitus. A prospective, randomized, double-blind, and placebo-controlled trial from Korea is presented evaluating the effect of intratympanic cortisone therapy in 30 patients with bothersome tinnitus [60]. 15 patients received intratympanic cortisone injection 4 times in 2 weeks, 15 patients received saline solution. Neither with regard to tinnitus severity nor with regard to loudness, could a significant difference between both groups be revealed although both groups observed an improvement of about 30% (evaluation 4 weeks after therapy).

#### 3.2.8 Muscle relaxants for tinnitus therapy

Baclofen, which is a drug from the group of muscle relaxants for treatment of spasticity in the context of spinal injuries, was applied in rats after acoustic trauma and in the animal model it led to a reduction of the tinnitus. Current trials in humans are currently not present [61].

Cyclobenzaprine is also applied as muscle relaxant for the treatment of skeletal spasms. Because of its analgesic effect, it was evaluated in 2 studies on tinnitus treatment. 65 patients were compared to 30 patients on a waiting list. 24% of the tinnitus patients had positive reactions on the relaxant that improved by 53% with regard to tinnitus intensity and 55% with regard to tinnitus induced distress [62].

In a multicenter trial (Regensburg, Brasilia), this agent was analyzed more exactly and thoroughly applied in several doses and compared to other muscle relaxants. Only a high-dose application of cyclobenzaprine (30 mg) tested in 14 patients led to a reduction of the tinnitus severity in the THI. For all other agents and low-dose cyclobenzaprine, no improvement was observed. The side effects were xerostomia, sleepiness, and constipation. The authors cannot explain the potential effect of the relaxant, however, they assume that it has a comparable effect with tricyclic antidepressants due to the similarity. They emphasize that only controlled studies with higher numbers of patients may provide reliable information on the potential effect in the context of tinnitus [63]. Trials with such substance groups are really only useful if they are randomized and placebo-controlled. Otherwise it seems that only the number of publications is important, new statements are not given by those studies. Furthermore, the rate of side effects is very high. In particular, there is not pathophysiological explanation that would justify the application of this drug in chronic tinnitus.

#### 3.2.9 Benzodiazepine and tinnitus therapy

Clonazepam and deanxit only cause low improvement. The study on the efficacy of benzodiazepine with an antidepressant conducted by the tinnitus group from Antwerp treated a total of 35 patients in 2 groups [64]. The study design was reported as being double-blind, randomized, and placebo-controlled, but this was only partly true. All patients were treated with the benzodiazepine derivative clonazepam, they were randomly assigned to 2 groups. Additionally, all patients of the first group received the antidepressant deanxit (melitracen, a tricyclic antidepressant drug) for 3 weeks, afterward the dose was continuously reduced. Then the patients received a placebo for 3 weeks. In the second group, the sequence was reverted, again with additional clonazepam. This application was double-blind. 7 patients interrupted the study so that 28 cases could be evaluated. Only 3 patients observed an improvement of their tinnitus after melitracen, none of them after placebo. The authors exclude a positive effect of clonazepam alone because the value of depression (in the BDI) was not improved. Finally, the authors postulate that it is reasonable to combine substances that have an effect on several neuro-transmitters. In summary, a reduction of the tinnitus complaints was reported.

This study on clonazepam combined with an antidepressant is methodically misleading because a substance combination was tested so that the term of “placebo-controlled” is not applicable because it only refers to one substance. Further it is not mentioned that the benzodiazepine clonazepam has a very high potential of addiction, disturbs the sleep rhythm, and influences the learning ability so that it should not be prescribed for therapy. A positive effect as postulated by the authors in their summary, can actually not be concluded.

In Korea, all tinnitus patients seem to be treated with benzodiazepines. In one study the spasmolytic clonazepam was randomized crossover with Ginkgo biloba (!). A total of 38 patients received this therapy (0.5 mg of clonazepam and 40 mg of Ginkgo each of them 4 times daily). Clonazepam in high doses reduced the tinnitus loudness (in 74% of the patients), the severity (79%), and also the annoyance measured in the THI (61%). Ginkgo biloba, however, did not show any effect. The conclusion of the study was that clonazepam was effective in tinnitus therapy [65].

Because of the high risk of addiction, the administration of clonazepam is controlled by the narcotics law already after short time of intake.

With the mentioned high dose of this agent, numerous symptoms disappear because the patient is stunned. Not only the tinnitus gets calmer, as promised in the title of the article, but certainly the whole patient. This article was actually rejected by the *European Archives of Oto-Rhino-Laryngology*, however, it appeared in another journal – good scientific customs!

#### 3.2.10 Antidepressants for tinnitus therapy

In an update of a Cochrane review from 2006 and again 2009, the study situation of the efficacy of antidepressants in tinnitus therapy was analyzed. 6 studies encompassing a total of 610 patients were evaluated. Only one high-quality study was found on the effect of a serotonin re-uptake inhibitor (SSRI), but it could not confirm an effect on the intensity of the tinnitus and its severity. Studies with tricyclic antidepressant were of lower quality and could not delimit the effects on anxiety and depression from tinnitus severity. In the meta-analysis the conclusion is drawn that there is still not proof that antidepressants improve tinnitus [66]. Nonetheless, antidepressants are often successfully applied in the treatment of chronic, decompensated tinnitus, however, not for improvement of the tinnitus but for treatment of accompanying depression and anxiety. If those conditions are improved, often also the tinnitus is perceived as less bothersome. So the question of the Cochrane analysis seems to be useless because nobody will receive and take an antidepressant without having symptoms of depression or at least sleep disorders.

In cases of according comorbidity, antidepressants are a useful completion of the therapy and lead neither to addiction nor do they sedate the patient in contrast to benzodiazepines, barbiturates, or anticonvulsants.

#### 3.2.11 Other substances

Ginkgo is not effective for tinnitus treatment. In 2012, a review article from Norway as update of a Cochrane review analyzed recent randomized and placbo-controlled studies on the effectiveness of ginkgo biloba with a total of more than 6000 patients. Evidence could not be revealed neither for the efficacy in the context of cognitive deficits, dementia, apoplexy, claudication nor for tinnitus. Moreover, even if mild, several side effects were observed such as vertigo, stomach complaints, or allergic reactions, and sometimes even increased bleeding tendency [67].

This Cochrane analysis was updated in 2013 [68]. Four new trials with more than 1500 patients were evaluated: in 3 trials, tinnitus was the main diagnosis; in the context of the 4^th^ study, patients were treated with ginkgo who suffered from mild to moderate dementia, some of these patients also had tinnitus. No efficacy could be proven in the patients with tinnitus alone, the dement patients with only little tinnitus distress observed a low improvement. The authors postulate that ginkgo is ineffective in patients with the main complaint of tinnitus.

Already in 2001, an article was published in the British Medical Journal (BMJ) on the effectiveness of ginkgo extract as treatment for tinnitus in a very large patient population (1,121 participants) assessed in a double-blind and placebo-controlled trial. Ginkgo did not lead to improvement of the tinnitus penetrance and intensity, neither did a placebo [69]. Numerous trials have confirmed the ineffectiveness of the ginkgo biloba extract for tinnitus treatment during the last years, the more thorough the study was, the clearer was the result [70]. Only the tiresome application observations and paid adverts in our journals as well as one “review article” always cited by the pharmaceutical industry [71] continue reporting the contrary. However, especially this review article excluded all above-mentioned trials – the reason for this aspect is not explained in the text.

#### 3.2.12 Gabapentin – treatment with neurotransmitters 

In an older analysis, gabapentin was applied for tinnitus treatment [72]. Gabapentin is an antagonist of gamma amino butyric acid, which is an inhibitor transmitter. 52 tinnitus patients received 1,800 mg gabapentin every day for 5 weeks, the control group consisted of 24 patients who received a placebo. Both groups had a light high frequency hearing loss. After treatment, significant differences regarding tinnitus severity were not observed between both groups.

#### 3.2.13 Zinc as tinnitus medication?

116 tinnitus patients, all of them older than 60 years, were treated either with 50 mg of zinc per day or with placebo in a randomized, double-blind, and placebo-controlled trial. After 1 month of interruption, the groups alternated [73]. Zinc is expected to promote the postsynaptic activity of some neurotransmitters, in higher ages, the concentration of zinc in the serum is generally lower. However, this study from Iowa did not reveal a positive effect on the tinnitus after treatment with zinc in comparison to placebo.

#### 3.2.14 Ozone and betahistine for tinnitus therapy

A prospective trial of 68 tinnitus patients from Turkey was performed in a randomized and prospectively controlled way [74]. In 10 sessions 27 patients received ozone as autohemotherapy twice a week, 26 patients received 48 mg betahistine per day for 3 months, and 15 patients in the control group received no therapy. Ozone is applied as anti-inflammatory agent and as complementary treatment against ischemia, betahistine is expected to have a vaso-active effect and thus improve the circulation of the inner ear. No significant differences could be revealed for none of the groups neither with regard to tinnitus severity nor to loudness.

Still pharmacotherapy in tinnitus disease is not very promising and has no proven evidence. Even the application of intratympanic cortisone cannot be justified for tinnitus alone and not at all for persisting tinnitus even if a certain – however rather temporary – placebo effect is observed. Zinc, ozone, and betahistine are evidently ineffective as well as ginkgo. This last aspect is very interesting and confirmed by a Cochrane analysis because especially in Germany the effect of ginkgo on tinnitus is propagated loudly, even for hearing loss and for prophylaxis of noise-induced damage it is promoted, preferably by life-long intake!

The German as well as the American practice guidelines come to the conclusion that there is no proven effect for any medication in the context of chronic tinnitus persisting for more than 3 months. A review article from Spain confirms this statement. The literature with regard of numerous pharmacotherapeutic approaches was assessed [75]: anticonvulsants, anesthetics, antidepressants, antihistamines, benzodiazepines, corticosteroids, as well as diuretics. In single cases success is reported everynow and again but the evidence is very low. At the same time there are other trials that do not confirm any effect for the same pharmaceutical product.

In this situation, experienced physicians who can refer to complex diagnostics and a good cooperation with audiologists and psychologist are responsible for the therapeutic effect. This was described by a review article from the USA [76].

In summary, there is still no sufficient evidence and no clinical experience for the effective application of pharmaceutical products in order to really and effectively treat or only suppress acute or chronic tinnitus.

## 4 Neuromodulation – instrument-based medical interventions

Since pharmacological solutions cannot be rapidly found in the near future, the basic conditions for effective tinnitus therapy are oriented rather on cochlear and central mechanisms. 

In his review article, Noreña from Marseille, France, examines all possible origins of tinnitus. They are found on the cochlear level, mainly in the external hair cells, and caused for example by noise trauma or stimulation peaks in the inner hair cells with activation of the NMDA receptors. In the central auditory system, tinnitus develops by aberrant activities that are mostly generated by plastic alterations caused by hearing loss, hyperpolarization of neurons of the thalamus, or a general increase of the central activity of the auditory system with increased influence of even non-auditory irritation [8].

In terms of therapy, this knowledge led to the development of different therapeutic approaches especially in the last years attempting to directly influence the central stimulation patterns or re-organization in the primary or secondary auditory cortex. A recent review article that was presented during the international tinnitus seminar in 2014 in Berlin, describes cortical magnetic stimulations, direct or indirect electric stimulations, but also attempts to influence acoustically those aberrant stimulation patterns [43].

The most important therapeutic approaches will be presented in the following paragraphs and assessed critically with regard to their evidence.

### 4.1 Inner ear (electro-stimulation – local anesthetics – neural therapy – laser)

In the past, there were many attempts made to directly reach and influence the inner ear. Because of lacking success, those ideas have not been further pursued.

#### 4.1.1 Cochlear electro-stimulation

Some groups examined the effectiveness of electric stimulation of the inner ear. 120 patients with tinnitus and hearing loss were divided into 2 groups in the context of a double-blind and placebo-controlled trial. 80 patients were stimulated via the auditory canal (non-invasive) with frequencies of 250–8,000 Hz for 4 minutes each and an intensity of 1.15 mA, 40 patients were placebo-treated, i.e. without electric current. This trial from Poland revealed that the tinnitus disappeared immediately after treatment in 33.6% of the treated patients and in 6.1% of the placebo group. However, the follow-up showed results that were less favorable but the improvement of the group of electro-stimulation was significant. The changes were assessed by means of a self-designed questionnaire encompassing 20 items [77].

In the context of a trial from Israel, 10 tinnitus patients were stimulated with an intratympanic needle electrode (comparable to the promontory test) and pulses of 100 and 1,800 Hz with amperages of 0–1 mA. The therapy was applied for 30 minutes each on 3 subsequent days. The tinnitus severity improved in 5 patients, but turned to the original status 4 weeks after treatment. Also the audiological tests could not reveal any changes – the authors postulate that this therapy might be useful for some patients; further it has no side effects [78]. The authors consider as effective a suppression of the tinnitus by the (peripheral) stimulation but not masking. This effect can only be temporary. Anyway, this non-randomized and in particular not placebo-controlled study is only an approach without any evidence.

#### 4.1.2 Local anesthetics

Direct injection of local anesthetics or diffusion into the middle ear by means of iontophoresis had no influence on the tinnitus, not even temporarily. Neural therapies with targeted injections “around the ear” can possible relieve contractions or even pains, however, the tinnitus cannot be influenced, neither in terms of loudness nor regarding its concrete bothersome distress. Current and especially valid studies were not published. Success rates published many years ago [79] could never be validated afterwards.

#### 4.1.3 Soft laser – no therapeutic effect on tinnitus

Only the approach that has been propagated for more than 30 years to irradiate the inner ear with a soft laser, i.e. non-cutting bundled light, and to thus influence the tinnitus, still has supporters, and especially sellers. Theoretically, the laser treatment is expected to stimulate energetically the inner ear, some laser therapists even claim to be able to treat hearing impairment [80]. At the end of the last century, some trials proved that each form of laser radiation (different wave lengths, with and without additional ginkgo) cannot effectively influence the tinnitus [81], [82], [83], [84]. Nonetheless, this therapy is still propagated in single cases and even an expensive self-treatment device is marketed (“Tinnitool”). Recent studies, however, confirm again the ineffectiveness.

In an analysis from Milan [85] 60 tinnitus patients assigned to two groups were treated either with active laser light (650 nm, 5 mW) or with a dummy in a prospective, randomized, and double-blind trial. All patients had concomitant hearing loss. The therapy was performed for 20 minutes per day over 3 months. The therapy success was assessed by means of the THI, a statistically significant difference could not be revealed between placebo and active laser irradiation. Also tinnitus masking and hearing capacity did not change, however, the tinnitus loudness measured in SL was slightly lower in the laser group.

The study is characterized by the fact that also the impaired hearing ability was measured – in all patients, beside other audiometric data. A therapeutic effect cannot be achieved by soft laser therapy, neither with regard to the tinnitus nor as hearing improvement.

In Iowa, a double-blind, placebo-controlled trial was conducted that evaluated the effect of laser therapy on hearing functions [86]. 30 patients were randomly assigned to 3 groups and either treated with low level laser (n=9), with placebo (n=10), or not at all (n=10). Laser and placebo treatment were applied in 7 steps to different points of the head for a total of 4 minutes. Before and after treatment, subjective audiometric data were assessed and otoacoustic emission were measured. In none of the groups, altered hearing functions could be revealed neither with nor without treatment.

In Europe and especially in Germany, low level laser therapy was heavily propagated about 30 years ago without being able to provide a proof of success. In the USA, it appeared in the last years, rather promoted for improvement of speech understanding. However, success could not scientifically be confirmed even if the presented study only treated very small numbers of cases and furthermore different regions of the head were “irradiated”. 

In a double-blind, placebo-controlled trial from Austria, 48 patients showed no significant therapeutic effect in comparison to placebo [87].

In 2014, 43 patients were treated in the context of a double-blind, placebo-controlled prospective, randomized trial in Malaysia. The laser device (“Tinnitool”) was actively applied in 22 patients, in 21 patients it was not turned on. Between both groups there were not significant differences. In the active group, 41% observed an improvement, in the placebo group even 59% of the patients [88].

In cases of noise-induced tinnitus, a study from Persia revealed an improvement for the first three months after 20 session with low power laser therapy, afterwards no effect could be found. There was no control group [89].

Laser therapy with a completely ineffective low power laser stimulator is in the market since more than 30 years. Success had never been proven, again and again there are attempts to establish this kind of devices and to sell them at high costs (“Tinnitool” costs more than 1000 Euro!). Meanwhile those commercial business models reach developing countries, however, also there they scientifically prove to be ineffective. 

### 4.2 Cortical interventions – transcranial magnetic stimulation

From the discipline of psychiatric treatment of depression, the idea was taken to influence tinnitus by transcranial magnetic stimulation. This therapy option for chronic tinnitus was evaluated more intensively during the last years, especially different stimulation frequencies and locations were tested.

The (psychiatry) group from Regensburg [90] initially assessed the effect of neuronavigated repetitive transcranial magnetic stimulation in chronic tinnitus on the tinnitus-induced distress while the cortical stimulation zones were determined in a methodically complex way and then stimulated specifically at low frequencies. 12 tinnitus patients with increased metabolic activation in Heschl’s convolutions of the cortex underwent radiation with 2,000 stimuli per day and a frequency of 1 Hz; the tinnitus severity could be significantly reduced. No significant improvement could be observed after placebo radiation with a so-called sham coil.

Another trial of this group treated 32 patients with repetitive magnetic stimulation either in a low-frequent temporal or high-frequent prefrontal and low-frequent temporal way. Directly after therapy, both groups showed improvements but 3 months later the group with the combined therapy had better results [91]. In contrast, Lee et al. from the USA reported about 8 patients who received rTMS on 5 subsequent days on the left temporoparietal side. A therapeutic effect could not be achieved [92] .

In a study from Washington [93], 14 adults (42–59 years) who suffered from tinnitus persisting for more than 6 months were treated with low-frequent (1 Hz) repetitive transcranial magnetic stimulation or a sham probe. The tinnitus severity was assessed by means of the THI. Real treatment improved the value of the THI by 5 points, placebo even by 6 points. Thus the treatment was not more effective than with placebo.

Also the group of Tübingen around Plewnia considered the success of rTMS therapy in a rather sober way: 48 patients were treated for 4 weeks, all of them improved a bit based on the tinnitus questionnaire which was observed after placebo as well as after active treatment [94].

On this topic, a current study from Utrecht was published with the conclusion that rTMS was ineffective for treating chronic tinnitus [95]. The randomized, double-blind, placebo-controlled design of the study enrolled 50 patients treated with rTMS (low frequency, 1 Hz or placebo). The evaluation was performed with the tinnitus questionnaire and the THI and VAS. At no time of the follow-up period a significantly better effect of real rTMS could be observed compared to placebo.

A recent study from Portland randomly treated 70 tinnitus patients from 2011 to 2014 on 10 subsequent days with active rTMS or placebo [96]. Side effects were not observed, 56% of the actively treated patients (n=32), but also 22% or the patients treated with placebo (n=32) had improved findings regarding tinnitus severity. The therapeutic success was stable during the 26 weeks follow-up.

#### 4.2.1 Magnetic stimulation and location of the coil

Regarding transcranial magnetic stimulation, a strong magnetic field is induced by electric current circulating in the coil. These magnetic waves penetrate the skull and cause an intracranial current reversal. The neuronal activity shall be manipulated by different stimulation variations. Many studies, however, differ regarding the location of the coil. It is placed on areas that are either marked by certain EEG alterations or pre-defined in the functional MRI scan, other studies stimulate always the same regions and vary in terms of stimulus intensity or alternate the stimuli.

A Chinese investigation of 22 patients (12 active, 10 placebo) determined the laterality of the auditory cortex with MMN measurements (mismatch negativity), 9 of 12 patients reported a reduction of the tinnitus after magnetic stimulation regarding severity and loudness, however, after 1 months it was no longer significant [97]. The abstract of this study promises more than the actual investigation contains. In the abstract a very promising therapy is mentioned, in the article, and only there, a short sentence is found that the effect was only short-lasting! This article was reviewed and published in a journal with high impact factor!

#### 4.2.2 Magnetic stimulation and different types of stimulus

Numerous publications from the group of Antwerp evaluated different stimulus intensities and rates in always very small patient populations. Only 1 Hz stimulation (applied in 11 patients) seemed to be effective with improvements of 20–40% regarding the tinnitus loudness. Long-term effects were not investigated [98].

Every now and again, the group of Regensburg presents results documenting a rather positive effect. 192 patients who received 10 stimulations over the left auditory or frontal cortex and who were compared to a placebo group (sham treatment) could not report significant reduction of the severity measured by the tinnitus questionnaire, however, the total effect in the follow-up was not significantly better than after placebo treatment [99].

Afterwards, the group assessed only those patients from their collective of 235 patients – a total of 50 patients or 21.3% – who reacted positively on the therapy. Even 2 and 4 years after treatment the results were better than before, however, they became worse in the further course [100].

In another analysis of 538 tinnitus patients, the group looked for predictors for successful treatment with magnetic stimulation [101]. Even if both stimulation types showed an improvement measured with the tinnitus questionnaire, the effect sizes were very low. Predictors for a successful treatment could not be revealed.

#### 4.2.3 Combined application of rTMS and antidepressant

In a study from Turkey, 5 groups containing 15 patients each were compared [102]. Beside a placebo group, 2 groups received transcranial magnetic stimulation with different pulses, 1 group received an additional antidepressant (SSRI), 1 group received only magnetic stimulation. In the follow-up time after six months only patients had an improved tinnitus severity (THI and TSI questionnaire) who had received the antidepressant, with or without rTMS. The data, however, were retrieved from only a very small group of patients which limits the significance – the combination of antidepressant and TMS does not seem to have a generally superior therapeutic success.

#### 4.2.4 Review articles on rTMS

Meanwhile also some meta-analyses are available for this therapy.

Two review articles from China and the USA document careful, rather positive effects of non-invasive magnetic stimulation for chronic tinnitus. The Chinese review article found 5 randomized controlled studies with a total of 160 patients but only short follow-up intervals. The authors conclude that long-term effects cannot be determined reliably [103]. An article from Boston assessed 105 publications with a total of 1,815 patients with special attention to side effects. The authors come to the conclusion that side effects are not exactly mentioned or documented. They are generally considered as mild, but occurred in 16.7% [104].

In a review article on repetitive transcranial magnetic stimulation studies were assessed until 2014 and 15 RCTs were evaluated [105]. Generally, the authors state that the studies cannot be easily compared because the numbers of patients were very small, different types and locations of stimulation were used, the rating of the success was different, and in particular no really adequate placebo stimulation was available. Up to now, there is no placebo procedure that the patient does not recognize as such. Nor any study could organize the blinding of the therapeutic staff. This relativizes the “success rates” and according to the authors they should be interpreted most carefully. Nonetheless, the authors state in their conclusion (and of course in the abstract) that rTMS was a therapeutic option, but with modest effect.

#### 4.2.5 Significant noise exposure in magnetic stimulation

An interesting issue which is known from diagnostic MRI, was dealt with by a group from Lyon. They investigated the noise exposure during magnetic stimulation. Significant sound levels were measured, depending from the stimulation intensity peak levels of 120–140 dB(A) were measured with a middle level of 90–100 dB(A). Further, it was interesting that the coil used in the trials as placebo (sham) was quieter of up to 40% [106].

This article confirms that ear protection should be worn during therapy, and it also tries to reveal a correlation between active stimulation and auditory-cortical reactions on noise exposure.

This study raises important questions regarding noise exposure by therapy and the simultaneously occurring cortical reactions on noise exposure alone. At the same time the sham stimulation is even more doubtful because this coil does not become warm and is much quieter – which actually nullifies the placebo effect.

#### 4.2.6 Can rTMS be effective?

An interesting and pioneering article was published by the research group from Groningen on the cortical plasticity with the question if hyperactivity of the auditory cortex occurs only in tinnitus patients or if it is a general cortical property. In the studies on transcranial magnetic stimulation, this hyperactivity is valued as correlate of the tinnitus. The cortical metabolism was measured by means of FDG-PET in 20 tinnitus patients and compared with 19 persons without tinnitus. In both groups, the metabolic activity in the left auditory cortex was higher than in the primary right one, for the secondary auditory cortex, the metabolic activity was reciprocal, on the right side higher than on the left. However, there was no difference between tinnitus patients and control persons; thus it seems that they will have to be considered as regular reaction of a healthy brain [107].

But the effects of rTMS on the cerebral function do not seem to be clear. The group from Regensburg analyzed the literature with regard to possible alterations of the motor cortex after different magnetic stimulations, but they did not find systematic and comprehensible alterations of the cortical irritability [108].

In a guideline from several European countries, the statement on repetitive transcranial magnetic stimulation is given that an analgesic effect on the primary motor cortex is evident (level A). For depressions, a level B effect could be assumed with probable effectiveness, but for tinnitus treatment only a level C effect could be observed (probably effective) [109].

In summary, magnetic stimulation (rTMS) is certainly a scientifically sound approach that is very complex and has its weaknesses with regard to studies since the patients can recognize if a coil is active or not (missing warmth and less loud) which makes placebo stimulation not real. For all therapy studies, higher case numbers and especially longer follow-up times must be required in order to really identify and prove long-lasting and effective therapeutic approaches for chronic tinnitus.

However, the results of rTMS are described in contradictory ways. While the group from Regensburg reports about therapeutic success with different application types and series for several years, data from the USA do not confirm these findings with still very few case numbers. But it is surprising that none of the numerous studies in this field includes audiological examinations. If magnetic stimulation changes cortical (or subcortical) reactions, measurable changes should be found for example in subjective central hearing tests or cortically evoked potentials and contingent negative variations. So only the subjectively perceived severity is the criterion of success.

In conclusion, it can be said that transcranial magnetic stimulation is a promising therapeutic option for many study groups, especially psychiatrist. However, it is effective only for short intervals and not superior to placebo. Often secondary result parameters are used in those studies (especially in trials from Regensburg and Antwerp) that reveal better success for the stimulation group. If the results are not sufficient, they are “corrected” (calculated in another way). In the past, this type of therapy was tentatively applied in psychiatric centers for treating depressions, in comparable studies with similarly poor success.

In summary, the very contradictory study results of the last years seem to show that rTMS for tinnitus is effective for the time of treatment but long-term efficacy cannot be confirmed. This statement is in accordance to the fact that the original hypothesis is not true that hyperactivities of the auditory cortex were pathognomonic for tinnitus. However, many treatment attempts are based hereupon with influencing the cortex by magnetic stimulation as well as directly or indirectly with noises (“neurostimulation”). Even if a recently developed European guideline confirms a possible (level C) effect, there are meanwhile sound studies like the one from the Netherlands [107] proving that transcranial magnetic stimulation has no effect on chronic tinnitus.

### 4.3 Electro-stimulation

Another possibility of directly influencing cortical structures consists of applying electrical stimuli. This aims at influencing aberrant electrical activity in the cortex. Assumed synchronicity could get out of the rhythm or the electrical stimulation could initialize counteracting stimuli that might then eliminate or influence the reaction of tinnitus. This stimulation can be applied transcutaneously or directly into the cortex, but previously it has to be clarified which region shall be stimulated. These therapeutic approaches have been used for many diseases in psychiatrics – all of them with rather poor success. Also for tinnitus treatment we look back to multiple investigations. Based on the knowledge that cortical alterations are at least responsible for the persistence of tinnitus, the attempt is made in many centers to stimulate the auditory cortex or other cerebral regions directly or transcranially by electric current.

#### 4.3.1 Transcutaneous, transcranial electro-stimulation

The efficacy of transcutaneous electro-stimulation for tinnitus treatment was evaluated in a small patient population (n=31) with placebo control. Low-frequent alternating current (<100 Hz) with intensities of 50–2,000 mA and a pulse rate of 30 Hz was applied. Significant improvement in comparison to the placebo group could not be achieved [110].

In a trial from Regensburg [111], 32 patients with chronic therapy-refractory tinnitus underwent bifrontal electro-stimulation with 1.5 mA for 30 minutes 6 times, twice per week each. The results were assessed by means of the tinnitus questionnaire. There was no improvement of the tinnitus severity, not even of the depression. Loudness and annoyance improved minimally based on subjective impression.

In a double-blind and placebo-controlled study from Belgium [112], 20 patients with chronic tinnitus received transcranial electro-stimulation for 20 minutes either anodically, cathodically, or with sham stimulation (placebo). During treatment, the intensity of the tinnitus changed, the severity was not assessed. Especially the anodic stimulation reduced the intensity of the tinnitus during treatment, some patients even reported an effect of several days.

The study group from Antwerp presented a study of 111 patients with tinnitus persisting for more than 1 year; 36 patients received transcranial direct current stimulation (tDCS), 37 had transcranial alternating current stimulation (tACS), and 38 received transcranial random noise stimulation (tRNS) (with randomly stimulating and changed alternating current voltage). Significant improvement (VAS tinnitus loudness and severity) was observed especially in the context of random noise stimulation (tRNS) [113].

These types of treatment and electric stimulations have certainly less side effects; the evaluation revealed some improvements immediately after stimulation, but longer lasting success could not be achieved or it was not even assessed. Furthermore, VAS and non-validated questionnaires are a weak instrument for evaluation.

A group from Switzerland investigated the effect of transcranial direct current stimulation in a double-blind and placebo-controlled study with 42 patients [114]. The cathode was placed on the auditory cortex, the anode in the prefrontal region. No side effects were observed, but there were neither effects on the tinnitus.

In 21 out of 26 patients (77.8%) from a New Zealand study, an improvement of the tinnitus loudness and severity of at least 1 point after 2 sessions of tDCS (different locations and intensities) was achieved [115].

Japanese researchers investigated the influence of the connectivity by tDCS [116]. Since the connection between the left and the right auditory cortex seems to be less developed in tinnitus patients in comparison to regularly hearing patients, 9 tinnitus patients were compared to 9 control persons. After tDCS, the connectivity between auditory cortex and somato-sensory and motor brain areas was reduced in tinnitus patients, while this connection remained strong in the control group. It is unknown why this study did not provide data on the hearing loss of the tinnitus patients so that no statement could be given on the correlation of hearing loss and tinnitus.

#### 4.3.2 Invasive cortical tinnitus therapy

Many tinnitus patients are ready to undergo even invasive procedures in order to get rid of their tinnitus. In an investigation from California, 800 firefighters were asked if they would be ready to undergo intracranial surgical radiation with the so-called gamma knife at the caudate nucleus to treat their tinnitus. The readiness depended on the prognostic success: in case of 100% success, 60% of the interviewed persons would agree to this therapy; in case of 75% success, there were still 43%. Below those values, the readiness was significantly lower [117].

#### 4.3.3 Intracortical implantation of electrodes

While transcutaneous electro-stimulation has no effect on the tinnitus perception according to the available publications, the question remains if direct intracortical stimulation is more promising.

At this point, an article from 2011 will be cited as example of very far-reaching therapeutic options. In Antwerp, electrodes were implanted in the secondary auditory cortex in 43 tinnitus patients who had previously responded positively on transcranial magnetic stimulation (rTMS) for a certain time [118]. Three days after implantation, the electrodes were stimulated with 40 Hz bursts. If no response was observed, the burst frequency was changed until a reaction on the tinnitus was perceived. The success was measured by means of a visual analog scale (VAS) before and after treatment, there was a placebo group but it could not be evaluated because of reasons that were not explained in the article. A total of 29 patients out of 43 responded to electro-stimulation, but only 8 reported a real improvement. Statistically, the VAS data revealed 67% success rate, 33% of the patients did not respond with regard to their tinnitus. In terms of side effects, 3 patients developed epileptic seizures, 1 patient had brain hemorrhage and consecutive difficulties with speaking and finding words (however, the tinnitus had improved), 1 patient developed a brain abscess that had to be treated surgically (and the tinnitus became worse).

Fortunately, such therapeutic adventures are very rare and probably cannot be carried out in our country because there are efficient and responsible ethical committees. Already the assessment of such invasive procedures only with VAS shows that a real evaluation is not performed. It remains unclear why the placebo group could not be evaluated and how it was stimulated. Finally the side effects and complications are mentioned but the authors still think that a good and potentially promising therapy is found.

The group from Antwerp used transcranial magnetic stimulation for exact definition of the location of implanted electrodes for tinnitus suppression (1 patient) [119]. A direct electro-stimulation of the auditory cortex led to significant reduction of the tinnitus in this patient who underwent implantation of an intracortical electrode. Another patient with extracortical electro-stimulation experienced a short-term, non-persisting tinnitus reduction. The implantation of electrodes into the defined region was carried out after intensive diagnostics with functional MRI examination of the tonotopic tinnitus representation in the cortex [120].

Another study with 8 patients who suffered from chronic tinnitus and who underwent neurosurgical electrode implantation after fMRI examination and electro-stimulation for 2 weeks, observed similar results of an at least partial tinnitus reduction. In this study, the stimulation was interrupted after 2 weeks and replaced by sham stimulation. In this period, no further improvement was found [121].

In a double-blind, placebo-controlled, and randomized cross-over study from Bordeaux, 9 patients with severe unilateral tinnitus underwent electrode implantation in the auditory cortex under general anesthesia [122]. This electrode was connected to a stimulator implanted in the pectoralis region. For 4 months, the patients were biphasically stimulated, afterwards there were randomly assigned to 2 groups and either placebo or really stimulated. After a washout they were again stimulated cross-over, therapeutic success was measured by means of a questionnaire (structured tinnitus interview – STI). One patients had to be explanted because of severe psychic decompensation; 3 patients were explanted after the end of the study, whereas 5 were stimulated for further 3 years. During the open phase, the tinnitus improved in 5 patients, 2 patients reported about deterioration of the findings. In the following control phase, also improvement was found, however, it was seen in the placebo group as well as in the directly stimulated group. Side effects were not observed, in particular no changes in hearing. The authors conclude from this investigation that direct electro-stimulation of the auditory cortex is associated with significant placebo effect, already because of the surgery. A therapeutic effect on tinnitus severity is not achieved in consideration of this placebo effect.

In summary, regardless the side effects, all those studies are not appropriate to confirm a valid and significant therapeutic success because of the low numbers of patients (n=2 or n=8 or 9), also because the placebo effect must not be neglected. Since electro-stimulation is perceived by the patient, even switching off the electrode is no real placebo. In addition, many articles do not mention possible risks and side effects.

#### 4.3.4 “Deep brain stimulation”

A recent review article from Maastricht shows possible therapeutic perspectives by deep brain stimulation for which an electrical pulse generator is implanted into cerebral structures (“brain pacemaker”). This therapy had positive effects in the symptomatic treatment of therapy refractory Parkinson’s disease and shall be evaluated for application in tinnitus treatment. It is based on the reflection that the stimulation zones in tinnitus patients are found in very different brain areas so that they would have to be stimulated individually [123]. Investigations on this topic are currently not available. Anatomically and surgically, such a targeted implantation of electrodes seems to be possible, if it turned out to be therapeutically reasonable in cases of tinnitus [124]. The German practice guideline on deep brain stimulation, however, does not include the indication of tinnitus [125].

Deep brain stimulation in humans has not been validly investigated with regard to tinnitus, because of its invasiveness this procedure has to be intensively and carefully discussed. In contrast, a well-designed study from Bordeaux [122] reveals that the invasive procedure of direct intracortical stimulation temporarily reduced the tinnitus severity in some patients, but that it is not better than placebo stimulation. It is surprising that no side effect occurred since other neurosurgeons observed severe complications after electrode implantation [118].

In the context of transcranial stimulation, which is significantly less invasive, no relevant effects with regard to tinnitus could be found in this methodically good investigation performed in 42 patients. Studies with an improvement of the severity by 1 point (!) and doubtful changes of non-confirmed connectivity are simply irrelevant. Furthermore, no data is retrieved in these studies on the hearing loss which would be essential for the evaluation of the connectivity. Again the question must be asked how so poor studies pass the review process and are published in reputed journals.

### 4.4 Vagus nerve stimulation

Transcutaneous vagus nerve stimulation is considered as new and quasi most recent type of tinnitus treatment. Medially highly appreciated and published in the journal *Nature*, those studies referred only to animal experiments until now.

These animal experiments led to the knowledge that animals learn more easily when the vagus nerve is electrically stimulated, simultaneously to the exercises [126].

For the treatment of chronic tinnitus in humans, the ear noise shall be modified by sound therapy that is accompanied by direct vagus nerve stimulation. In a pilot study on the general feasibility, 24 tinnitus patients underwent vagus nerve stimulation for 3–10 weeks and were monitored regarding cardiac complications in order to explore clearance and safety of this therapy. Two severe cardiac complications occurred, the therapy seemed to shorten the QRS complex in the EEG, in primarily healthy persons, arrhythmia did not occur [127]. Another feasibility study was based on the question if such a treatment was really possible in humans. In New Zealand, 10 tinnitus patients were selected and electrodes were implanted at the left vagus nerve at the neck. The patients heard noises for 20 days for 2.5 hours per day that did not correspond to the tinnitus frequency. At the same time the vagus nerve was electrically stimulated. The therapy was well tolerated. Side effects did not occur. According to the authors, this means that this therapy is safe and feasible [128].

In general, a stimulation of the vagus nerve leads to a reduction of the sympathetic innervation which was proven by an article from Leeds evaluating 48 healthy test persons [129].

A multicenter study on vagus nerve stimulation that was initiated in the USA 2 years ago is currently not finished or even published. The homepage of the company does not reveal recent statements even if they are expected to be present. So only a case study from New Zealand was presented until now (and published in a highly ranked journal) [130]. In a 59-year-old patient who suffered extremely from his tinnitus and who had undergone – unsuccessful – intracortical implantation of electrodes, the vagus nerve stimulator was implanted and he received sound therapy for 4 weeks. The achieved improvement (measured by THI) and also the improved depression persisted for 2 months, afterwards the severity of the findings was the same as before therapy. Of course, the authors postulate that the vagus nerve stimulation could become an effective therapy.

It is really astonishing that de Ridder and his team from (now) New Zealand and his colleagues from Antwerp seem to be able to publish in highly ranked journals without any problem even if the scientific output of their studies is more than doubtful and from one single case that has to be considered as unsuccessful they deduct positive therapeutic effects although those were not persisting and even failed.

### 4.5 Trigger therapy and acupuncture

#### 4.5.1 Tinnitus treatment at myofascial trigger points

Myofascial trigger points (MTP) are characteristic for patients with myofascial pain syndrome. Possible relations to tinnitus modulation were investigated in a study from Brazil. Often the MTPs are found in the postural muscles of the neck and the shoulders. In this study, 94 tinnitus patients were examined with regard to MTP, then the myofascial trigger point was palpated. In 68 tinnitus patients, but only in 34 participants of the control group of the same size without tinnitus, at least 1 MTP was discovered. Half of the tinnitus patients with an MTP could modulate the tinnitus by stimulating this point [131].

This study has parallels to the somatosensory system and tinnitus perception. The authors rather see an interrelation to pain syndromes where the tinnitus is an accompanying symptom of myofascial pains and tensions. Further, the modulation of the tinnitus experience does not necessarily mean an improvement. However, the attempt to detect those MTP in tinnitus patients seems to be reasonable, especially when patients report about trigger points and influence on the tinnitus.

#### 4.5.2 Acupuncture 

For the first time, studies on the effectiveness of acupuncture in tinnitus are presented that are randomized and qualitatively sufficient. A randomized, double-blind study with sham control from Korea assigned 33 patients with unilateral tinnitus and mild to severe hearing loss either to a real acupuncture group or to an alleged acupuncture group where no meridian points are used. The treatment was performed in 10 sessions (twice a week). The THI and VAS were assessed at the beginning of therapy and 3 months later. The THI score improved with low significance after 3 months only in the real acupuncture group, in the other one no changes were observed. Also the data collected with VAS showed improvements for the real acupuncture group, however rather low. The authors conclude long-term effects from real acupuncture [132]. In contrast, a meta-analysis from Korea comes to the conclusion that the investigated 9 RCTs were of low quality. From 382 articles, 373 had to be excluded, mostly because other therapies were combined with acupuncture (phytotherapy, biofeedback, other medication). In 5 studies, acupuncture was compared to sham treatment, however, without statistically significant changes. Two studies had compared acupuncture with pharmacotherapy and achieved improvements in the tinnitus patients with acupuncture who had a primarily psychogenic tinnitus. The authors of this review article conclude that there is no convincing evidence for the success of acupuncture in tinnitus treatment. Nonetheless, acupuncture would be an option because of the few side effects and the missing alternatives, if the patient was not open to psychological therapy [133].

The first study encompasses only a small number of cases, the success is low, and the follow-up time only amounted to 3 months. The cited meta-analysis included this study, however, the results were valued as not being significant with regard to actual tinnitus severity. In summary there are numerous studies but all of them applied acupuncture only as additional option. Sound and high-quality studies are rare and also do not confirm a therapeutic success of acupuncture in tinnitus treatment. The low rate of side effects and the calming, tension-resolving effect are in favor of acupuncture treatment.

A group from Denmark investigated the effect of electro-acupuncture for treatment of chronic tinnitus [134]. A total of 50 patients (46 male, 4 female) with chronic tinnitus were randomly assigned to 3 groups and treated either with manual acupuncture, electro-acupuncture, or placebo. The tinnitus penetrance, intensity, and effect on the quality of life were assessed at the beginning, after 6 treatment sessions, and 1 month later. Six treatment sessions with 8–10 acupuncture points were performed by an experienced acupuncturist. There were no significant differences between the 3 groups. The authors conclude that acupuncture is not effective for tinnitus treatment.

Acupuncture is often recommended for tinnitus treatment as procedure without side effects although there are not hints at all for an effectiveness (in tinnitus) as it is also confirmed by the cited Danish study with however a small number of cases.

### 4.6 Acoustic stimulation – modulated sounds

#### 4.6.1 Tinnitus therapy with external noises or frequency modulation

Masking is considered as the oldest effective tinnitus therapy. Already Aristotle (384–322 BC) wondered why the humming sound in the ear stopped when someone made another noise and suggested that it was probably because the louder noise chases away the lower one. Aristotle referred to his own tinnitus that could be covered or masked by the sounds of the sea at the seaside (cited according to Feldmann [135]).

Also modern therapeutic reflections include active hearing. Since tinnitus is a symptom of disturbed hearing perception and since at the same time the brain is able to perform plastic remodeling processes in every age of life, it seems to be reasonable to use the plasticity to habituate the interfering noise of the tinnitus or to filter it from the active perception. This is only possible when hearing is made aware and also gets a high significance. For this purpose, the daily hearing situations have to be experienced consciously, deficits have to be recognized, and further hearing strategies have to be learned. In this context, masking is only one variant of possible approaches. Since sounds are eliminated or at least influenced by other sounds, multiple acoustic stimulations have been introduced in tinnitus therapy.

It is a problem that often devices are developed and then sold expensively, especially because there is a big market and patients often look desperately for “THE solution”. Such therapies exist since many years, they come and go because generally no scientific proof can be achieved confirming persistent improvement by tonal stimulation. Often acoustic therapies are promoted by stating that alleged changes in the brain of the affected and then treated patients are documented, mostly by EEG or MEG measurements. Thus, additional seriousness is pretended that is rarely proven – in particular these kinds of documentation are only snapshots that are valued as therapeutic effects without performing follow-up examinations and individually transferable regular values.

“Neuromonics” therapy from the USA and Australia uses tinnitus-specific acoustic stimuli. The acoustic stimulation first took place with sounds, later the company switched to musical stimulation with alienation in the tinnitus frequency. Here, several studies are found that are all not convincing. The device itself (available in the USA) is distributed by audiologists and it is rather expensive.

In an investigation from Florida, the effect of this so-called Neuromonics Tinnitus Treatment system (NTT) was evaluated. However, beside specific acoustic stimulation, also counseling and support by an experienced therapist for tinnitus treatment was performed [136]. The stimulus is a spectrally modified broadband musical sound that is adapted to the individual hearing loss of each ear. In the multicenter study, 52 patients with bothersome tinnitus were examined, 51 patients with a mean age of 55 years were evaluated. The patients did not have relevant psychosomatic comorbidities (depression, anxiety), the tinnitus severity was defined by means of THI. The treatment consists of two steps. During the first 8 weeks a broadband, individually adapted noise was included in music, the patient got used to it and adjusted the volume to a comfortable level. The second phase took 4 or more months, the patient heard the music without the broadband (masking) stimulus, possible for 2–4 hours per day. Also relaxing and breathing techniques were taught during treatment. 77% of the patients reported about significant improvement in the THI. The authors mentioned the limitations of this study because there was no randomization and no placebo control. The therapeutic success improves by using music instead of simple and often disturbing noise because music itself often has a pleasant and relaxing effect.

A group from Cleveland compared the treatment of commonly used sound generators with the Neuromonics device. In total, 56 patients were treated, 23 with conventional noise generators and 33 with the Neuromonics device. Therapeutic success was evaluated with questionnaires (THI). Both groups had significant success whereas those patients had better results who previously had more severe findings. Because of economic reasons, the treatment with conventional noise generators should be preferred according to the authors because the Neuromonics device was much more expensive. Nonetheless the special interest and preference of the patients should be considered [137].

Beside musical stimuli and acoustic stimulation, the sound therapy with NTT also uses elements of TRT such as counseling and care so that it is effective because of this multimodal approach. This is why this therapy does not fulfill the criteria of real evidence.

#### 4.6.2 Acoustic neurostimulation

Especially in Germany, the treatment with acoustic neurostimulation based on the so-called CR procedure (coordinated reset), patented by the inventor and partial distributor, was introduced and massively promoted since 2009. The company made use of an interesting business model and distributed devices (which were MP3 players preset on the exactly measured tinnitus frequency) via associated ENT practices that could participate in the total price of around 3,500 Euro. The introduction and in particular also medially offensive promotion took place without that even reliable therapeutic results were present. Poster and congress presentations depicted merely high success rates of the treatment, at the beginning even hearing improvement by this therapy was announced (however, those “results” were no longer mentioned in later articles). The articles that were published from 2012 on, never achieved an evidence degree.

The theoretical idea of the so-called CR (coordinated reset) therapy is based on a model consisting of the main precondition that tinnitus develops because – possibly due to missing input from the periphery as in cochlear hearing loss – neurons fire synchronously in the primary auditory cortex and connect to other brain centers (“connectivity”). The model uses other models of influencing brain activity and neuronal networks, is mathematically deduced and refers actually to electrical stimulation causing an elimination of the neuronal synchrony in a reset and thus an asynchrony. But now it is suggested for tinnitus treatment to apply this stimulus acoustically in an exactly calculated paradigm in relation to exactly measured tinnitus frequency via a headset. Preferably, the period of the stimulus should exactly correspond to the EEG delta activity via the temporal flap. The acoustic stimulation should, as known from hearing aids where the tinnitus frequency is superposed by the enhancement, desychronize the tinnitus [138].

The first study [139] presented on this topic evaluated 63 patients with chronic (>6 months) tinnitus and a maximal hearing loss of 50 dB in the stimulus frequencies. They were randomly assigned to 5 groups, the smallest group consisting of 5 patients underwent placebo treatment, and the other groups received acoustic stimulation of different degrees for 12 weeks. The device was applied every day for 4–6 hours (groups 1–3) or 1 hour per day (groups 4 and 5). While groups 1–4 were stimulated in the frequencies of the previously (exactly) measured tinnitus frequency with 4 or more sounds, the placebo group was stimulated with low-frequent sounds (300–600 Hz). Group 1, of which the stimulation algorithm was expected to be effective, consisted of 22 patients, the other groups had 12 patients. In some patients, additional EEG was performed twice; among them 12 patients were selected for exact evaluation of the EEG. For measuring the therapeutic success, the tinnitus severity and loudness were evaluated by VAS and the tinnitus questionnaire according to Goebel and Hiller [24]. In 2 of the groups, the VAS score improved significantly, also compared to the small placebo group. Also the questionnaire scores improved significantly. Finally also the tinnitus frequency was significantly reduced (about 28.5%). Regarding the EEG measurements, an increased alpha activity and reduced delta and gamma activities were obtained in the 12 selected patients, the type of stimulation is not clearly described in this article. A total of 15 adverse effects were reported but not described, 13 of them were therapy-related.

At first sight, both articles seem to be extremely sound from a scientific point of view with numerous formulas for model calculation e.g. of the stimulation frequencies. For example, the neuronal natural frequency of the tinnitus patient (how is it measured?) and the synaptic interaction are included in the formulas.

For statistical evaluation, a “Euclidean distance formula for cluster analysis” is used but with the soft values of visual analog scales. The whole therapeutic algorithm is based on the hypothesis that tinnitus develops by neuronal synchrony which of course is then “proven” by therapeutic success in 22 patients. However, it remains unsaid how such a neuromodulation and influencing of neuronal synchrony can take place only by sounds that are perceived via the inner ear being mostly impaired in particular in the frequency of the tinnitus and that then have to pass the whole auditory system.

In general, based on animal experiments with direct electrical stimulation this therapy is now performed in humans. However, the direct electrical stimulus on the neurons is applied indirectly via the auditory system that is no longer intact.

At least, some of the patients of this study had hearing loss (max. 50 dB), but differentiation and assignment of the results was not performed. Fortunately, the study describes that improvement of the hearing threshold could not be measured (improvement was reported in previous results), but the tinnitus frequency reduced of up to 28% (!). In the discussion, these findings are explained with the Zwicker tone as consequence of modified inhibition. But why does the inhibition change the frequency and in particular what is the benefit for the patient? Especially the link to the EEG measurements that are “clearly pathologic” indicates a very mechanistic understanding of the disease – finally the EEG pathologies are treated. The connectivity of the brain functions is emphasized but it is expected to be interrupted only by desynchronizing the neurons in the primary auditory cortex. However, in the context of tinnitus, connectivity means that the acoustic perception is combined with emotional valuation and reactions. And these only change if the tinnitus disappears permanently and completely. When the tinnitus loudness is reduced or only its frequency changes, there is no long-term effect. Furthermore, there was no real validation of the results based on a sufficient follow-up time of this study.

In addition, it is a main precondition for the exact setting of the acoustic stimulation that the tinnitus frequency is measured exactly in the frame of 500 Hz. For unexperienced patients who are not musically trained and who further suffer from hearing loss, this is very difficult especially for high frequencies, sometimes even impossible. The patented assignment of the stimulation sounds, however, becomes rather arbitrary considering this inexactness. Another crucial weakness of this study is the fact that the participants had to pay for the device. For scientifically valid studies this is an impossible requirement because study results, especially imprecise visual analog scales might be falsified by economic reflections.

Due to this really weak and often criticized study, first application observations in ENT practices were performed without control groups and with the possibility for patients to buy the device at a lower price. Furthermore they were refunded part of the expenses when they had completed the study, but they had no return right. Then the University of Nottingham initiated a scientifically sound, placebo-controlled study that was completed in 2014. The results of this trial were held back by the distributing company that supported this study. Up to now, it is not allowed to publish the trial. However, at least the therapeutic results are meanwhile published after several legal quarrels and made available to parts of the public, e.g. support groups. According to the results, the effects of real CR stimulation are as good (or as bad) as placebo therapy. In Germany, the company is meanwhile insolvent but a successor company tries to replace the device in the market.

In summary, it is certainly positive to treat tinnitus with acoustic stimulation, especially when it takes place in the frequencies of the hearing loss and the ear noise. However, this is better performed with hearing aids and additionally improves the communication. If hearing aids are not indicated, music or natural sounds are more comfortable (and less expensive) than patented sounds.

#### 4.6.3 Sound and noise therapy

Sound therapy with different sounds was evaluated in 2 randomized trials of a total of 70 patients with chronic subjective tinnitus. Pure tones in the area of good hearing were used as well as pure tones in the frequencies of the hearing loss, and harmonically complex sound in the region of the hearing loss. All patients observed improvement, there were no significant differences between the various types of stimulation. The authors conclude that the effect is less based on the acoustic stimulation alone but rather on the concentration and active focus on hearing [140].

In another study (double-blind and cross-over) pure tones with and without phase shift were applied in a total of 22 patients. For none of the stimuli, a significant effect was observed [141].

In another investigation 20 patients were treated with amplitude and frequency modulated sounds. Amplitude modulated sounds in the frequency of the tinnitus led to a slight temporary suppression of the tinnitus in 90% of the patients; long-term effects were not evaluated [142].

#### 4.6.4 Meta-analyses on acoustic stimulation

A Cochrane meta-analysis [143] evaluated 6 trials with a total of 553 participants and generally stated that no improvement of the tinnitus could be evidently measured after applying external sounds alone or their enhancement (by hearing aids). However, the analyzed studies mention that sound therapy was helpful and supportive; a clear definition of the evidence was not possible because of the generally multimodal therapeutic approaches. It is emphasized that in contrast to numerous pharmaceutics such a therapy had not adverse effects. The authors explain that the lack of clear evidence does not contradict to clinical effectiveness and importance of this therapy; generally the therapy of chronic tinnitus is a combination of several treatment approaches. 

The group from Nottingham around Hoare and Hall presented a review article valuing different treatment approaches with external noises for tinnitus treatment [144]. Generally, these applications are based on the idea that tinnitus modifies the tonotopic map of the cortex and an increased spontaneous activity of the neurons of the auditory system is present, accompanied by emotional interconnections. Different approaches of sound therapy are described in this review article, as for example acoustic neuromodulation, stimulation with tones individually set according to the tinnitus (“serenade”), with music modified in the tinnitus frequency (“neuromonics”), with hearing aids and special relaxing sounds (“Widex Zen”), or a personalized auditory training with sounds. According to the authors, for all these methods of acoustic stimulation, only very few evaluable studies are found. Evidence, even weak, exists only for special auditory training programs that are based on personalized frequency training. Other relevant studies, e.g. on acoustic neuromodulation, are announced but they could not be published because of missing success (see above).

Sweetow from San Francisco criticizes sound therapy and the missing evidence of many studies [145] because they generally do not consider sufficiently the concomitant hearing loss. He presents new music-related therapy approaches in terms of interrupted sounds. They are presented via hearing aids, take into consideration the hearing loss, and at the same time they have a relaxing effect, but they are not associated with known and emotionally connoted music.

Sound and noise therapies are part of several habituation approaches such as tinnitus retraining therapy (TRT), often they support psychotherapeutic interventions. In general they are not sufficient as only device-related therapy and lead to additional frustration of the patients because these treatments are often expensive and not paid by the health insurances. The review article from Nottingham objectively describes this situation and requires or initiates even valid studies. This applies to merely sound-based procedures such as neuromodulation or other sound therapies and also to individual attempts with tinnitus tones. The Cochrane analysis confirms that until now there are no investigations that prove a clear evidence for this therapy alone, even if it is often an important part of the whole treatment concept. 

### 4.7 Music therapy

If inhibition and filtering of background noises have to be re-learned and consolidated by plastic modifications in the cortex for effective habituation of the tinnitus, hearing has to be intensified and made aware. To understand hearing in this quasi new dimension, is particularly successful when the artistic type of acoustic stimulation, the music, is used. In the music, all rudimentary hearing experiences are implied, at the same time experiences develop by listening to music that may be touching and influence emotions. 

Among many therapeutic approaches, music is firmly established, but nearly always as part of multimodal treatments or as completing therapeutic option.

In single cases, special music therapy was developed for tinnitus and successfully applied in clinical practice without that controlled studies are presented [146].

#### 4.7.1 Heidelberg music therapy

In the last years, a group for music therapy from Heidelberg developed a special program for music therapy for patients with chronic tinnitus and published according studies.

The Heidelberg model of music therapy is based on the concrete quality of the tinnitus frequency (according to audiologic diagnostics) of the patient and embeds this acoustic sensation in musical experience. This therapy is accompanied by intensive information, counseling, and training of relaxation techniques. In single therapy sessions, the attempt is made to influence the hearing perception as a whole with the objective that patients are enabled to control effectively the perception of their tinnitus. Singing and working with a gong are oriented at the tinnitus perception, singing is performed “around” the tinnitus tone.

In the first study [147], a relatively small patient population was enrolled (n=20); in 7 of 10 patients music therapy led to a clinically relevant improvement while only 2 patients of the control group that was not treated by music showed this effect. These values were also stable in the catamnesis. The authors especially emphasize that this kind of tinnitus therapy takes the patient from his passive role, which cannot be achieved for example by instrument-based procedures (noisers).

In another study, high therapeutic effects (80% improvement) are reported [148]. A follow-up study was carried out in the context of which 206 patients were contacted and 107 questionnaires could be evaluated. According to them, 76% observed an improvement in the tinnitus questionnaire after treatment [149]. Furthermore, the Heidelberg music therapy was applied as additional option to pharmaceutical treatment of acute tinnitus in 23 patients, also here, according to the authors, the patients’ findings could be improved by the accompanying treatment [150].

In an MRI study of 20 patients with acute tinnitus (compared to 22 untreated patients) changes were found that consisted of an increase of grey matter in the auditory cortex and the frontal brain regions of the treated patients [151]. At least, the authors mention that their patients were only mildly impaired and the measurements of the grey matter are not very precise. Furthermore, the patients had acute tinnitus that is often characterized by relevant spontaneous recovery.

Nonetheless, the Heidelberg music therapy – actually a therapeutic approach encompassing counseling, relaxation, habituation based on music – is scientifically doubtful because the studies are uncontrolled. Success is attributed only to music therapy although all elements of counseling, relaxation, and finally also psychic stabilization would have to be considered. In particular, the catamnestic data must be questioned and many non-responders are not mentioned or not included in the evaluation. The current practice guideline on tinnitus [31] does not certify evidence for this music therapy.

In contrast, real music therapies, receptive or active, are certainly useful and effective in single cases, however, no evaluated studies are available. These music therapies must then be considered as special type of psychotherapy.

#### 4.7.2 Tailor-made-notched-music (TMNM)

Comparable to the way of the Neuromonics device, the group around Pantev from Münster developed a therapeutic procedure where tinnitus patients hear music that was lowered in the tinnitus frequency – which is expected to promote inhibitor effects already after very short listening time [152]. For the treatment, the tinnitus frequency is exactly measured, then the patient selects his (preferred) music out of which the tinnitus frequency is filtered – quasi shall the tinnitus complete the music in this way.

In the first studies from 2010, patients with chronic tinnitus were treated [153], [154]. An evaluation was performed in 10 patients with a tinnitus of <8 kHz and 10 patients with a tinnitus of >8 kHz. Treatment took place on 5 subsequent days for 6 hours every day with this special music that was modified in the tinnitus frequency (tailor-made-notched-music training, TNMNT). The 2 study groups had no differences regarding age and hearing loss. Only the patients with a tinnitus below 8 kHz observed an improvement immediately after therapy and 30 days after therapy. The improvement concerned the tinnitus severity as well as its loudness, however, the loudness changed only for a short time and even increased after therapy. In contrast, the patients with high-frequent, over 8 kHz ear noise had no improvement. The authors explain these findings with the poorer sensitivity of the cochlea in high frequencies and the simultaneously observed higher hearing loss for those frequencies – so an adequate stimulation in those frequencies is not possible with regular volumes.

In another study on this therapeutic approach, the cortical plasticity was intensively described and particularities of inhibition and habituation were discussed with regard to musicality and musical promotion. Therefore 39 regularly hearing tinnitus patients (frequency of <8 kHz) were randomized and double-blinded in 3 groups (therapy, placebo, and listening to music without editing). In the therapy group the tinnitus frequency was measured and the area of one octave around this frequency was filtered out. In the placebo group, frequencies below and above the tinnitus were filtered out. The patients listened 1–2 hours per day to their individually preferred music for 12 months. The results revealed changes of the tinnitus loudness and a reduction of the cortical activity in the MEG only in the treatment group [155].

The authors describe this therapy as support of the rehabilitative plasticity. A particular difficulty was the exact and especially the reproducible definition of the tinnitus frequency, which is not easy for musically unexperienced people. Furthermore, a significant hearing loss must not be present, the study set a limit at 35 dB, because the stimulus frequencies actually have to be heard. Additionally, TNMN was combined with tDCS, i.e. direct current stimulation. 32 tinnitus patients were stimulated in different ways (cathodic and anodic stimulation or sham-placebo) and in parallel, they listened to the specific music therapy for 10 days. There were no differences regarding tinnitus distress just due to this additional therapeutic element [156].

A completing article from Münster investigated the neuroplastic modifications of specific tinnitus music therapy and compared them with patients who actively make music. Only patients who listened to this especially edited music observed improvements, not the ones who do not actively make music [157].

The group from Münster has now introduced an enhancement of 20 dB (spectral energy contrast, ISEC) in addition to the filtering of music around the pre-defined tinnitus frequency and compared 18 patients (9 with and 9 without ISEC). In both groups the tinnitus loudness was reduced. Magnetic encephalography revealed that the according neuronal activity was reduced in both groups, the ISEC group even had higher inhibitor effects [158].

Already at the end of the 1980ies, the so-called “Tinnicur” treatment tried to modify relaxing music with pass filters in the tinnitus frequency and to thus modify the ability to filter and the inhibit tinnitus perception. The efficacy of this therapeutic approach could not be proven. Instead of editing the music in the tinnitus frequency, INMN attempts to filter exactly this frequency. However, this means that the tinnitus has to be exactly defined where only very experienced examiners succeed, especially when the patients are not very musical. The numerous, partly fMRI and MEG controlled music therapy studies turn out to be effective, however, they only encompass very small numbers of patients. The tinnitus has to be below 8 kHz and no significant hearing loss has to be found so that actually regularly hearing patient with tonal tinnitus were investigated, which is certainly only a very small percentage of tinnitus patients.

If this therapy is additionally compared with active musical work, it remains completely unconsidered which plastic changes and thus also promotion of the inhibitor abilities and the cortical hearing processing is achieved by making music. Effects on tinnitus severity can never be assessed immediately, nor confirmed by MEG measurements because an exact analysis of the MEG changes that only originate from tinnitus depends largely on the interpretation. Postulated changes in 9 patients cannot be considered as generally valid. It is not possible to define tinnitus severity if mechanistic models are developed in order to understand such a complex phenomenon!

Currently, this therapy experiences a real hype because clever companies with technicians used the idea from Münster and developed an innovative smartphone app (“Tinnitracks”), however, independently from the researchers from Münster. In this context, it is problematic that patients have to define their tinnitus frequency alone which is naturally accompanied by a high failure rate. But since an app is always considered as innovative, this company already received an innovation award, so this product will be self-propelling – at least for a certain period of time.

So it is clear that the group from Münster tries to diversify its approach. However, it can be doubted if the acceptance increases by the applied increase of the marginal frequencies. An according device was already in the marketplace before the study had been published. In this study, the test persons were not obliged to pay, the authors want now to develop cheaper solutions with common CD players and test them in larger patient populations. However, comparable to neurostimulation with pure tones around the tinnitus frequency, the complete hearing system and thus also the primarily often damaged inner ear is used for sound perception. The deficits of the auditory system influence the cortical inhibitor effect, furthermore the tinnitus frequency has to be measured exactly which is often very difficult in the high frequencies.

Even if procedures with modified music adapted to the tinnitus are certainly effective only in a limited way, they have at least an accompanying emotionally stabilizing and relaxing effect.

### 4.8 Hearing aids and cochlear implants

In the majority of the cases, tinnitus is accompanied by hearing loss and is mostly in the frequency of the hearing loss. Hereby the hearing loss is not necessarily perceived as impairing or severe, the distress is rather caused by the tinnitus [159]. For plastic restructuring of the auditory cortex, it is reasonable to stimulate especially the missing frequencies in order to propel inhibition, to suppress an increase of the limit frequencies and to restore the normal tonotopy of the auditory cortex. This is particularly successful with modern hearing aids that work well also in high frequencies.

That is why hearing aids certainly belong to the most effective tinnitus therapies which was confirmed by a prospective data collection from England. 2,153 patients were examined regarding their tinnitus severity. 1,440 received hearing aids. The improvement of the tinnitus penetrance by the hearing aids was significant, measured with visual analog scales. The effects with digital hearing aids were better than with analog ones [160].

A current study investigating what influences a patient’s career found out that the early application of hearing aids has to be promoted. A group from Mannheim followed-up 28 patients with “fresh” tinnitus for 6 months [161]. At first examination, an audiogram was made, the tinnitus loudness and the sensitivity to noise were measured with analog scales. During these 6 months, the tinnitus loudness and also the severity remained constant while the sensitivity to noise decreased. The initial depression as well as the hearing loss correlated with the higher tinnitus loudness. The authors conclude that an early detection of the depressive part is as important for the improvement of the symptoms as also the early provision of hearing aids.

Hearing aids are nowadays much better accepted than noise generators, as revealed by an investigation from Iran with 974 war veterans [162]. All of them had a clear hearing loss, 84% preferred hearing aids. Only 2% preferred noise generators, the remaining percentage used both devices. The use of hearing aids significantly improved the tinnitus severity.

A study from Milan [163] measured THI values before and 9 months after therapy. The patients received counseling and were provided with hearing aids for noise enhancement. The scores could be significantly improved (from 54.2 to 28.3 points). In a second part of the study [164], the effect of TRT with noise generators was compared to the effect with hearing aids in 91% of the patients. The study was carried out prospectively with parallel design, the hearing loss in both groups was moderate, but comparable. After the study, both groups observed significant improvement, independent from noise generators or hearing aids.

The cited studies do not give clear statements because it is a classical TRT design where psychosomatic comorbidities are not considered. Furthermore, the hearing loss was not exactly documented or assessed in a differentiated way, e.g. by hearing tests with background noise or tests for measuring central hearing performance. According to own experiences, modern digital hearing aids, with and without additional features, can be perfectly applied for tinnitus therapy. Especially since even patients with high-frequency hearing loss and highly frequent tinnitus may be treated due to open, very well tolerated provision with external receiver and good suppression of feedbacks and background noises, an important step forward was made. Those hearing aids can also influence highly frequent types of tinnitus because high frequencies are better transferred.

A group from Marseille reported about 74 tinnitus patients who received hearing aids with linear frequency transposition [165]. Those hearing aids are particularly active in high frequencies and thus appropriate for patients with steeply sloping. In 60 patients, the tinnitus could be permanently suppressed, 38 were examined more intensively: The majority (23) had tinnitus after noise exposure, the tinnitus suppression started few days after regular wearing of the hearing aid and persisted, however, a few days after removing the hearing aid the effect decreased and returned after re-application. Furthermore there was no dependence on the frequency of the ear noise. The authors explain this phenomenon that is only achieved due to the special transposition in the hearing aid with a reactivation of deprived areas of the auditory cortex. This leads less to a direct stimulation but rather to an opening of neuronal canals (gate mechanism).

In Auckland, a special setting of hearing aids for influencing the tinnitus was tested in 25 tinnitus patients. Single frequencies were downregulated and others were enhanced [166]. Most appropriate seemed to be a downregulation of 6 dB at 2 kHz. The according software shall be further investigated and correlated with the tinnitus frequency.

The principle of frequency transposition turned out to be useful in the adaptation of hearing aids in high-frequency hearing loss with steep decrease. Some producers of hearing aids proceed in this way for several years, especially in the context of fitting hearing aids in children in order to enhance speaking. Hereby, high frequencies are transformed and transposed to the mid-frequency range and then offered via the hearing aid. Other manufacturers try to maximally enhance the high-frequency range itself. Interestingly, a suppression of the ear noise is successful especially in tinnitus patients with high-frequent ear noise, however, this effect cannot be predicted for all patients. The French study confirms this circumstance in its patient population. Own clinical experience achieved similar effects via the other treatment option. Anyhow, the reactivation of the tonotopic map of the auditory cortex leads to plastic modifications that influence the tinnitus, either by direct re-stimulation or by a mediated gate effect.

Unfortunately, studies with hearing aids can merely be carried out, already because of the generally multimodal therapeutic approaches – finally the hearing aid itself is associated with diagnostics and information/counseling so that these elements represent a therapeutic effect.

In contrast to the application of noisers, a sufficient fitting of hearing aids reduces the stress of auditory processing and leads to cortical restructuring that might reduce the tinnitus and also hyperacusis. 

A recent Cochrane analysis emphasizes that hearing aids are usually part of a combined therapy so that they cannot be evaluated in an isolated and solitary way. There was only one study comparing the effect of hearing aids with the adaptation of noise generators that found out that both approaches had the same (positive) effect. All other studies stated a good effect of hearing aids but their adaptation was embedded in general multimodal therapeutic approaches [167].

Indeed, this is a deficiency: in up to 95%, tinnitus is associated with hearing loss and thus a symptom of disturbed hearing perception. In many patients, hearing aids achieve very effective relief, but there are no studies on hearing aids as isolated therapy, already because they are always encompassed in an audio-therapeutic total concept, which is confirmed by the Cochrane analysis. Nonetheless, hearing aids must not be neglected in the context of tinnitus therapy because they include and compensate hearing loss that is nearly always observed. In this way, cortical compensatory reactions and a decreased inhibition are reduced. 

#### 4.8.1 CI significantly improves stress caused by tinnitus in most patients

What is true for hearing aids, is even more valid for cochlear implantations. In hearing impaired or even deaf persons, a reactivation of the auditory cortex leads to modified stimulation patterns, to reduced spontaneous activity and thus to an enhanced inhibition of the auditory system for background noise. In many cases, but not in all, this leads to a suppression of the ear noises or at least to a reduction of their intensity.

An improved tinnitus severity in uni- or bilaterally deaf people by cochlear implantation is only a side effect but a very welcome one for the affected patients. A group from Groningen evaluated 212 implanted patients by means of specific tinnitus questionnaires (THI, THQ). 51.3% of the patients had tinnitus prior to surgery, in 55.6% of the cases, it disappeared or at least decreased after the intervention. However, the ear noise deteriorated in 8.2% of the patients and 19.6% perceived a new ear noise after implantation, which did not severely impair the patients [168].

Similar results were described by a group from Korea that evaluated the data of 35 CI patients [169]. Twenty-two (62.9%) had tinnitus before surgery, mostly the elder patients (>40 years). After implantation, the tinnitus was completely suppressed in 10 patients; all patients had better THI scores, i.e. reduced tinnitus severity. Also the tinnitus loudness measured with visual analog scales was improved, even more in patients >40 years. All patients were deaf in both ears and received CI on one side. So it is quite astonishing that this study describes improvement in all patients although they were only unilaterally treated.

The ENT Department of Ferrara [170] presented an evaluation of 51 bilaterally deaf patients; 36 of them additionally suffered from tinnitus. They received CI between 2005 and 2007. 36.1% of the patients reported that the tinnitus was completely suppressed due to CI; 41.6% observed a reduction. Also the severity was reduced in 75% of the patients, the THI was reduced in 72.2% of the cases.

A Canadian group from Ontario [171] came to similar results in 142 deaf people. Twelve months after CI, the severity of the tinnitus was clearly and significantly reduced. A complete suppression was successful in 37% of the patients, in 29% the tinnitus was reduced.

The group of the Charité, Berlin, Germany [172], presented a very extensive investigation where patients filled out standard questionnaires on tinnitus severity, stress management, depression, and anxiety as well as quality of life before and after cochlear implantation (for bilateral deafness or severe hearing impairment). Beside an improved hearing and speech comprehension, also a better quality of life was described after CI. The evaluated 39 tinnitus patients observed an improvement of their tinnitus symptoms. It was interesting that the intensity of the tinnitus before CI did not correlate with the evaluations on the quality of life and the psychosomatic comorbidities, however, it did afterwards. In a later investigation, the CI effects especially on tinnitus severity, stress management, and coping strategies were analyzed. Thirty-two patients with bilateral postlingual deafness who received CI were evaluated by means of several questionnaires. Twenty-eight of them had tinnitus prior to CI surgery, the others were tinnitus-free. After 24 months, 2 patients had no more ear noise, in 7 patients the tinnitus symptoms were unchanged (neither was the distress). In the other cochlear implanted patients, the distress and also the coping strategies had improved, also the quality of life improved of more than 50% in all patients [173].

The same group could then prove that the quality of life could be further improved by a second implantation, also with regard to tinnitus [174].

A similar investigation from Switzerland came to comparable results in an evaluation of 174 patients who received cochlear implantation: 71.8% had tinnitus before surgery, 6 months after surgery it had disappeared in 20% of the patients, in 51.2% it was improved. No changes were observed in 21.6%, 7.2% even reported deteriorated symptoms. Furthermore, 5 of the previously tinnitus-free patients had ear noises after surgery, those patients had developed also only poor speech comprehension [175].

In a prospective study of 38 patients from the Netherlands, the tinnitus severity could be significantly reduced by CI (uni- or bilateral implantation) (of 71.4% to 80%, according to the evaluation instrument). Five bilaterally implanted patients and one unilaterally implanted patient developed tinnitus after surgery [176].

#### 4.8.2 Even in cases of unilateral deafness, central hearing and tinnitus are improved

An extensive evaluation of 11 unilaterally deaf patients (mean duration of deafness of 25 months) was presented by the University Hospital of Freiburg [177], [178]. Twelve months after cochlear implantation, the speech comprehension with background noise was significantly improved compared to conventional CROS hearing aids and also BAHA as well as untreated hearing loss. Furthermore, 5 of those 11 patients observed complete and 3 partial tinnitus suppression 6 months after surgery. The 11^th^ patient reported that the tinnitus did not change when using the CI, but after switching it off, the intensity increased. 

Similar results were presented from Antwerp [179]: 26 patients with unilateral deafness and tinnitus were treated with CI. All 26 observed improvement of the tinnitus; the subjective loudness decreased (from 8.6 to 2.2, VAS). The scores of the tinnitus questionnaire were significantly reduced. The effect of the ear noises was independent from the tinnitus quality, narrow-band noise, tonal or even polyphonic tinnitus reacted in the same way.

A study that is more focused on tinnitus reports about 20 patients who were deaf in one ear and complained about very distressing tinnitus on the deaf side. Eleven patients had regular hearing on the opposite side, 9 patients had hearing aids. All patients received cochlear implantation on the deaf side. In investigations on speech comprehension and directional hearing, improvements could be achieved by CI also regarding hearing capacities. The loudness of the tinnitus was significantly reduced by the electrical stimulation of the CI [180].

The patients of this study had tinnitus and unilateral deafness for an average of 8 years, which importantly ensured a sufficient reaction on stimuli of the deaf side. In this study it could be shown that also the speech comprehension improved at least partly by CI and binaural stimulation.

A meta-analysis [181] evaluated 9 studies regarding the question of treatment of unilaterally deaf patients where an improvement of the tinnitus severity was achieved in 26 of 30 patients (87%), while 4 remained unchanged, and none of the patients observed poorer results.

Comparable to hearing aids, also cochlear implantations influence the cortical plasticity by reorganizing missing representations and thus interrupting the tinnitus maintenance. More complex questionnaires reveal that the deaf patients are more severely impaired by the whole situation than by the tinnitus alone. Only after CI, the tinnitus gets an independent significance – and is often very well suppressed.

The general discomfort of patients with unilateral deafness, who suffer very often from tinnitus, is significantly increased. Often the distressing tinnitus that cannot be suppressed or reduced with hearing aids leads to the decision to try with cochlear implantation. But tinnitus itself is never the only indication for surgery even if it is often the main reason. Some studies could additionally show that also the quality of life and the stress management can improve. The effects on ear noises are also positive in the majority of CI users. However, there seem to be patients who do not respond on electrical stimulation regarding the ear noises. A very small percentage of the CI candidates even develops ear noises after surgery.

If tinnitus justifies surgical intervention medically or economically, remains worth discussions. At least, habituation therapies are available that are effective also in unilaterally deaf patients. CI only reduces the loudness of the tinnitus and thus the impairment by the ear noise, but it does not completely eliminate the tinnitus. This discussion will certainly continue.

#### 4.8.3 Electro-stimulation of the hearing nerve

In an investigation from California, special electrical stimuli (biphasic with a defined stimulation rate of 100–200 or 5,000 st/sec with comfortable loudness) were tested in 13 CI patients in whom the tinnitus was only sometimes or never influenced by the CI. The patients used CIs of different manufacturers, the stimuli were generated with a special processor of the respective company. Nine of those 13 patients (69%) responded positively on at least one of the tested stimulation types, i.e. the tinnitus was partially suppressed. Significant differences regarding the stimulation rate, position of the electrodes, or loudness were not observed. The authors state that there are “responders” and “non-responders” among CI users, which means that some patients do not observe any changes regarding the tinnitus neither by CI nor by particular stimuli targeted to the tinnitus [182].

### 4.9 Hearing and audio therapies

During the last years, a hearing and audio therapy was developed in specialized centers that supports habituation of the tinnitus beside an improved treatment with hearing aids and especially the acceptance of hearing aids in older hearing impaired patients [183]. In this context hearing exercises are applied – the patient learns to blind out the tinnitus from the acoustic surroundings as an unimportant stimulus and to focus on other acoustic sensations.

An overview about the current status of hearing therapy and its history is given by Ptok et al. based on a research of the literature [184]. The modern hearing therapy originates from Urbantschitsch who promoted independent hearing exercises especially of hearing impaired patients. Only with CI technology and the clearly advanced hearing aids, hearing and audio therapy could be further developed in the last 30 years, and today it is a fixed part of multimodal tinnitus therapy and should also accompany the fitting of hearing aids.

According to a study from England, the units of hearing therapy were more effective when they were not too long. In a study with 48 participants, 4 groups were trained for different durations. The best group was the one that had the shortest therapeutic units, additionally, the success was best in the first sessions [185].

Hearing therapy is meanwhile defined in a manual [186]; it proved to be effective as symptom-related therapeutic option in behavioral therapy for tinnitus and hyperacousis in multimodal treatment approaches, however, valid data and thus evidence is only found for acoustic stimulation therapy but not for concrete exercises on improving hearing perception, because a clear separation from other therapeutic elements such as relaxation and focusing or defocusing is not possible. Furthermore, specialized centers cannot provide randomization or placebo therapy.

## 5 Habituation and cognitive therapies

### 5.1 Habituation therapies

Since several years, the therapy of chronic ear noises is performed successfully and scientifically proven with measures supporting habituation.

As multi-factorial disturbance of increased activity or missing inhibition in different centers of the auditory system, the chronic tinnitus is often associated with highly emotional, psychosomatic overlay.

Effective outpatient therapies for chronic tinnitus support the habituation of the tinnitus based on restructuring and relearning processes: The patient shall no longer consciously perceive the ear noise that is generated in the auditory system, most frequently in the inner ear. Habituation therapy with accompanying psychotherapeutic care can lead to important success. Music therapy and especially hearing therapies reasonably complete the treatment. In cases of chronic tinnitus, the use of hearing aids is certainly effective since it is often associated with hearing loss or is a consequence of hearing impairment. A review article about therapies that are especially promoted in Germany and scientifically evaluated comes to the conclusion that an integrative neuro-otological and psychosomatic therapy with particular emphasis on hearing therapy is effective as outpatient, in very severe cases even as inpatient therapy [187].

### 5.2 Therapy on the internet or by telemedicine

A new approach is tinnitus therapy via internet by manualized programs with and without therapeutic counseling (via telephone or e-mail).

A review article from Canada [188] reports about different approaches of internet-based tinnitus therapy, which can be started in different stages: in the diagnostics and evaluation only limited statements are possible because many questions need audiological competence and further audiometric examinations. Follow-up and (online) post-therapeutic care are possible and have been discussed in numerous publications. Approaches of internet-based behavioral therapy have also been developed and are currently validated, however, they are not sufficient for severely affected patients. Nonetheless, due to the high cost pressure the authors see an opportunity for (cheaper) internet-based care of tinnitus patients.

However, if diagnostics and therapy via internet are the solution for cost-related problems, can certainly be doubted; possibly milder cases may be treated in this way. Many patients have to be imperatively examined by specialists, already in order to evaluate an appropriate differential diagnosis. It will certainly be possible to perform subsequent examinations and follow-up controls via internet.

### 5.3 Tinnitus retraining therapy (TRT)

In 1996, Jastreboff and Hazell presented the tinnitus retraining therapy [189], [190] which is established since many years in outpatient centers worldwide and with high variability. At the same time, according studies especially from the Anglo-American area are often not controlled and are presented with only very moderate evidence.

In a contribution to the International Tinnitus Symposium in Berlin, Pavel Jastreboff gave a summary after more than 25 years of retraining therapy [191]: in more than 100 articles, significant improvement can be found in more than 80% for this therapy. TRT works with counseling in order to explain to the patients that tinnitus corresponds to a regular neuronal stimulation. The accompanying sound therapy (with noisers, hearing aids, and background noise) is expected to reduce the tinnitus-related neuronal activity. Therapy has developed in that way that especially negative assessments of noises (misophonia), and associated reflex-like reactions are processed in specific therapy protocols. The duration of treatment has reduced by these protocols.

#### 5.3.1 Tinnitus retraining therapy – stable success for a longer time

A study from Italy, controlled the therapeutic results for a total of 36 months. 45 patients with idiopathic tinnitus for more than 6 months were treated for 18 months according to the TRT scheme of Jastreboff (ENT-specific examination, audiological tests including DPOAE, intensive counseling, application of noisers and hearing aids as well as follow-up examinations and counseling). The therapeutic success was measured by means of THI. At the end of the therapy, the devices were further used; the patients had better results regarding concentration, sleep, relaxing, and social contacts, the hyperacousis remained unchanged. The THI score decreased of 20 points. Even 18 months after therapy, the values further improved. Especially the time after therapy is important because in 67% of the patients the tinnitus had not improved after 18 months of TRT. But in the time afterwards, the patients could better cope with the tinnitus due to improved relaxing and social activities and were less stressed [192].

#### 5.3.2 Tinnitus retraining therapy – effects on tinnitus-induced stress and loudness with unilaterally adapted noisers

In a Japanese investigation, 184 patients were evaluated of which 95 were treated only with TRT, i.e. intensive directive counseling and unilaterally adapted noisers [193]. After 6, 12, and 24 months, the according questionnaires (THI) were evaluated again. On average, the score decreased to 36 after 6 months and remained stable for 24 months. The additionally assessed stress level showed that more stressed patients benefited more from TRT than less stressed patients. The authors described – even if no control group was included – a positive effect of TRT, however, it was reduced by an existing hearing loss especially in the middle frequencies.

In a study from Illinois [194], the effect of TRT was compared to a control group that received only a minimal therapy consisting of diagnostics and counseling. 43 participants were included in this study after assessing 500 applications. The patients were randomly assigned to TRT (n=21) or the control group (n=22). However, 5 patients from the TRT and 6 patients from the control group had to be excluded so that finally 16 participants were in each group. Regarding the age (52–55 years), gender, tinnitus severity, and hearing loss, the groups were identical. The improvement of the tinnitus severity, measured with the THI, was very good in the TRT group with an effect size of 1.13 after 18 months, but it was also clinically relevant in the control group with 0.78 (an effect size of >0.2 corresponded to a low effect, >0.5 to a moderate, and >0.8 to a high effect). The authors concluded that TRT with directive counseling and noise stimulation is an effective treatment but also counseling alone is effective. Therapy might reduce the severity but not the tinnitus loudness.

In Belgium, a TRT study was performed treating 46 patients according to the classical retraining therapy of Jastreboff and Hazell. One particularity was that sound therapy was continued with a noiser even at nighttime during sleep. According to subjective patient evaluation, 80% improved, 20% observed no improvement. Patients with psychosomatic comorbidities had previously been excluded from the study [195]. A control group did not exist.

Tinnitus retraining therapy (TRT) in its classical form with directive counseling and sound therapy is more effective than without sound therapy, but in many US American studies, the psychic comorbidity is not assessed or patients suffering from depression and anxiety are not included in the studies. Thus, the effectiveness of this therapy only refers to a small patient population that in particular is less distressed. The evidence of those studies is limited because of small case numbers (e.g. 500 patients were screened, only 32 were included) and high failure rates (perhaps because of psychic comorbidities?). Further it is always astonishing how rarely an accompanying hearing loss is documented and therapeutically considered although the studies are carried out by audiologists.

#### 5.3.3 Tinnitus retraining therapy and noise generators (noiser)

A paradigm of classical retraining therapy according to Jastreboff and Hazell is the use of binaural noisers that should be set in their intensity below the tinnitus loudness, in the so-called mixing point. Masking of the tinnitus, however, impedes a tinnitus habituation. Tyler and co-workers from Iowa [196] randomly assigned 48 tinnitus patients to 3 groups: counseling alone, counseling with bilateral noisers completely masking the tinnitus, and counseling with noisers set at the mixing point. After 12 months, 3 of 18 patients of the counseling group had significantly improved results, 4 of 11 patients of the masking group, and 6 of 19 patients of the mixing point group. The mean improvement in the THI was 16.7% for counseling alone, 31.6% for TRT, and 36.4% for total masking. The authors concluded that masking leads to habituation in the same way as noise set at the mixing point.

In German speaking countries, noisers play only a subordinate role in the current therapy, even retraining. More often modern hearing aids are applied in order to treat often only low hearing loss in high frequencies. Indeed, some patients rather use noisers for masking or at least for distraction from their tinnitus. A dogmatic reflection in this context seems to be in vain as confirmed by the study of Tyler.

Certainly, TRT is a sufficiently effective therapeutic option for patients with less impairment, i.e. not decompensated patients. The study from Iowa additionally shows that the significance of noisers is rather limited, more important is the acoustic stimulation itself and especially the experience of the patients to be able to set something against the annoying tinnitus. Maskers as well as noisers, however, are contraindicated in cases of hearing loss, which is not considered in many studies, because they make understanding additionally difficult, enhance the stress caused by the hearing impairment and thus also the cortical counter-regulation in the sense of an increased spontaneous activity and reduced inhibition.

#### 5.3.4 Tinnitus retraining therapy – good therapeutic success can be achieved on an outpatient basis by using psychological approaches

In 2000, the ADANO (Association of German-speaking Audiologists and Neuro-Otologists) published a statement on TRT [197] recommending this therapy, but only as interdisciplinary treatment of ENT specialists, psychologists, and hearing care professionals (cited according to [159]). It was meant to emphasize that decompensated tinnitus requiring treatment imperatively needs psychotherapeutic counseling or therapy. During the last years, a successful, modified tinnitus retraining therapy was introduced at the Charité, Berlin, and scientifically accompanied leading to good results and at the same time high evidence.

From the outpatient tinnitus center of the Charité, 192 patients were treated outpatiently with this tinnitus retraining therapy and compared with a control group. TRT was conceived as multimodal therapy, completed by the technique of progressive muscle relaxation (PMR), physiotherapy, lectures, active redirection of the attention, and cognitive behavioral therapy. Therapeutic success was measured with the tinnitus questionnaire, the values of 45 patients on the waiting list served as control. From the outpatient initial treatment with follow-up care lasting for 7 days, resulted a significant short-term as well as persisting improvement of the tinnitus severity and other psychic disorders [198].

The study group of the tinnitus center of the Charité, Berlin, reported about long-term results of this tinnitus retraining therapy [199]:

In this pre-/post-study the data of 92 patients were presented who were treated on an outpatient basis with the German modified retraining therapy for one year. The treatment consisted of history taking, neuro-otological diagnostics, intensive and directive counseling, hearing aid, relaxation therapy, and psychosomatic counseling or therapy. The tinnitus severity was assessed by means of the tinnitus questionnaire of Göbel and Hiller. Only 9.9% of the patients had regular hearing capacities, the majority of 54.6% had a high-frequency hearing loss. 48.9% received bilateral noisers, 28.3% received hearing aids. The tinnitus severity decreased significantly after 3 months of therapy from 48 to 41 and after 6 months to 38 points, afterwards it re-increased by 1 point. Decompensated patients benefit best from this treatment, if indicated they received additional psychotherapy. Patients even benefited from the additional provision of hearing aids or noisers.

In 2015, again 130 patients had a follow-up examination 3 years after the onset of therapy [200]. The psychometric data were assessed at the beginning of therapy, immediately afterwards, and 3 years later. In summary, the quality of life of the patients had clearly improved after 3 years, the tinnitus severity, susceptibility to stress, and depression had decreased.

TRT is still a good treatment option for moderately affected patients. In cases of – very often observed – psychosomatic comorbidity, directive counseling and sound therapy alone are not sufficient. In many TRT studies, the therapeutic results are not validly measured to that their significance is reduced. The modified Berlin therapy takes these aspects into consideration and additionally offers psychological care – with very good success and especially with stable results for 3 years.

### 5.4 Hypnosis 

#### 5.4.1 Hypnosis therapy for tinnitus – doubtful benefit

An analysis from Cambridge evaluated studies about hypnosis therapy for tinnitus that were published in a peer-reviewed journal [201].

Since more than 30 years, success rates of up to 70% are reported for hypnosis, but the study situation is rather poor, especially with regard to the applied techniques. For some patients a positive effect is confirmed, but the evaluated studies cannot make clear if this success is due to general relaxation and stabilization effects.

Ross et al. [202] treated 393 patients with hypnosis in the context of an inpatient stay. The patients were treated in groups of 8–12 persons for 28 days in individual and group sessions. Therapy consisted of information, psychosocial counseling, muscle relaxation, music therapy, and Ericksonian hypnotherapy with single counseling. The patients were examined initially and 6 and 12 months after treatment by means of the tinnitus questionnaire of Goebel [24]; the results were compared to patients from a waiting list. The authors found clear therapeutic success. Nonetheless, the success of this multimodal therapy is certainly not only due to hypnosis. Furthermore, hypnotic procedures depend largely on the practitioner.

### 5.5 Cognitive behavioral therapy, other psychotherapeutic interventions

Since many years, therapies that are based on habituation and that – as recommended by the ADANO in 2000 – include psychotherapeutic elements besides informative counseling and hearing therapy are better evaluated and confirmed regarding their effectiveness. 

For German-speaking countries, an outpatient tinnitus coping program and training for hyperacousis was presented by Pilgramm et al. [203] in a review article without mentioning data from a study. The same applies for hearing therapy for tinnitus and hyperacousis [186] that proved to be effective as behavioral therapeutic, symptom-related therapy option for tinnitus and hyeracousis in multimodal therapeutic approaches, nonetheless no isolated data exist for concrete exercises aiming at an improved hearing perception (see above).

According to all meta-analyses, cognitive behavioral therapy seems to have a high evidence, surprisingly more with regard to tinnitus severity than psychic symptoms. In fact, accompanying depression (that is often found in tinnitus patients) is responsible for worse therapeutic results or non-response to therapy. Furthermore, cognitive behavioral procedures are nearly always combined with relaxation techniques, i.e. they are multimodal, without actual control groups (a waiting list is not really a reliable control group which becomes obvious in the context of a study from Sweden (see below)!). And of course there is no comparable placebo therapy. In terms of tinnitus therapy as mere cognitive behavioral therapy, there seems to be the problem that a disturbed hearing perception that certainly influences the tinnitus as symptom is neither assessed nor therapeutically considered. Since psychologists and psychotherapists generally perform this therapy, this cannot be expected. However, tinnitus is primarily an otologic symptom and should thus be treated by ENT specialists. An involvement of psychologists is useful but rather for treating psychosomatic comorbidities such as depression and anxiety.

However, when assessing therapeutic studies about tinnitus, it has to be stated that the best evidence is found for cognitive behavioral therapy.

#### 5.5.1 Cognitive behavioral therapy for tinnitus – meta-analyses

A meta-analysis from Sweden [204] evaluated the effectiveness of cognitive behavioral therapy (CBT) on the reduction of tinnitus severity. Fifteen studies were assessed with a total of 1,091 participants. Cognitive behavioral therapy had the most significant effect sizes compared to the control groups (passive = waiting list, active = other interventions). Later investigations confirmed the good effect sizes for tinnitus severity, a bit lower was the subjective mood of the patients. The authors conclude that CBT is effective for tinnitus treatment, however, long-term effects could not be finally assessed.

The same authors from Sweden [205] analyzed 11 studies where patients on waiting lists were defined as control group. A total of 314 patients were on the according waiting lists that lasted for 6–12 weeks. Considering these participants, there was a little but significant improvement of the tinnitus severity of 3–8% only because of the presence on a waiting list for therapy.

#### 5.5.2 Cognitive behavioral therapy – evidence for improvement of the quality of life, but not for loudness of the tinnitus or accompanying depression

An article from England investigated the effectiveness of CBT for chronic tinnitus in a Cochrane meta-analysis [16]. Six studies were analyzed with a total of 285 participants. Regarding the improvement of the tinnitus loudness, there was no significant difference compared to the control group (waiting list) after CBT. Even the comparison to other therapies did not reveal any difference. An investigation of the subsequent effects did not find significant improvement regarding depression in comparison to waiting lists and other interventions. The quality of life of the patients, however, was significantly increased by CBT in comparison of the other two groups which could be confirmed in other single studies, recently by Robinson et al. [206] who could show that there is an effect, but “only” regarding the quality of life. Of course, the tinnitus loudness does not improve by CBT. At least at first sight it seems to be surprising that concomitant depression is not improved after psychotherapeutic treatment.

However, considering the design of the analyzed studies, this contradiction can be explained as well as the limited significance. The participants of the investigation came from the outpatient, less affected group of patients after having agreed to a mostly publicly offered option. So they are probably different, as also criticized by McFerran and Baguley [207], from most of the tinnitus patients. In contrast to the inpatients, they should not have relevant psychic impairments, which is at least a double selection.

Under those special conditions, the patients – who were already previously motivated for the therapeutic procedure – achieved an improvement of their symptoms, but of course no relevant improvement with regard to a non-existing comorbidity.

In no case, a claim of superiority of cognitive behavioral therapy must be deducted from the cited meta-analysis of studies with very limited study design regarding its implementation in outpatient single therapy or daily clinical practice [17].

It remains scientifically unsatisfactory that representatives of psychodynamic therapies have not presented any evaluated investigations on the evidence of tinnitus going beyond casuistics [18], [19], [20].

Especially more affected tinnitus patients with a high level of suffering the psychosomatic sequelae are the part actually requiring therapy. The analysis makes clear that cognitive behavioral therapy alone is not sufficient in many tinnitus patients but should be accompanied by neuro-otological therapeutic approaches.

In a comparative study from Maastricht, 492 Dutch tinnitus patients who were previously untreated were randomly assigned to either a group treated with a special cognitive behavioral therapy as psychologically conducted single anamnesis and group therapy with sound therapy according to TRT or to a group that was treated with “regular” therapy. The regular therapy consisted of diagnostics, audiological anamnesis and evaluation of the complaints as well as application of hearing aids or masker. The following treatment encompassed audiological examinations and checking of the assistive hearing devices. In a second step, more severely affected patients were supported by a social worker for one hour in the “regular” group, in the cognitive behavioral group a 2-hour group therapy was initiated or psychological single therapy in cases of particular indication. Considering all assessed parameters, the special treatment was significantly more successful, in particular the quality of life increased whereas the tinnitus loudness and severity decreased. The treatment success was clearly more relevant regarding long-term effects, i.e. after 8 and 12 months it was more distinct than after 3 months. The authors consider the combined, interdisciplinary procedure especially successful even if they cannot clearly state which of the therapeutic elements contributed most to the total success. A subsequent study comparing the costs of those therapies was carried out as well [208].

According to this investigation, such a therapy is significantly less expensive: 626 patients were accompanied, the specialized, multidisciplinary tinnitus therapy was more successful and even cheaper than “regular” therapy [209]. In a recent review article, 31 studies on the effectiveness of cognitive behavioral therapy for chronic tinnitus were assessed. The results confirm that cognitive behavioral therapy is currently the best evidence-based therapy for chronic tinnitus. The analysis also confirms that multidisciplinary approaches are more effective [210].

In a study from the Department of Psychology of Marburg, 95 patients with chronically decompensated tinnitus were treated in single sessions with cognitive behavioral therapy [211]. Biofeedback assisted muscle relaxation was included in this therapy. The therapy lasted for an average of 19.9 weeks, the treatment was performed in a total of 3–4 diagnostic sessions and 12 strictly manualized treatment hours. At the beginning of the treatment hour, a neurofeedback-controlled relaxation phase took place followed by 15–20 minutes of cognitive behavioral therapy. Finally, a biofeedback training and again a short relaxation phase were performed for 20 minutes. The assessed data were tinnitus severity (tinnitus questionnaire) as well as other psychic symptoms (special questionnaires). 70% of the patients had concomitant disorders, 30% were depressive. In total, the tinnitus severity significantly decreased due to therapy. 74 patients (77.9%) responded to the therapy, 21 (22.1%) did not. For the therapeutic success, factors such as demography (age and sex) and tinnitus (duration, frequency, loudness) did not play a significant role, but the psychic comorbidities. So in particular depressive patients could not improve by this therapy. Audiometric data and thus hearing loss were not assessed and not considered in this study.

The data and thus the confirmation of evidence of therapeutic procedures that are currently applied successfully on an outpatient and inpatient base for habituation and stabilization are still rather weak. Since those procedures are mostly used in multimodal approaches, a controlled prospective study is scarcely possible, in many cases the control group consists of the waiting list that already has (because therapy is expected) a therapeutic effect. The same is true for cognitive behavioral therapy that – according to a Cochrane analysis of 2007 – provides evidence of the effectiveness for depression and anxiety (of tinnitus patients!) but not for the treatment of tinnitus. Indeed, the networking and interdisciplinary centers have to initiate studies that have exactly defined control groups. A comparison with a group that is called “usual care” as in the cited study from the Netherlands is too undefined and general. However, this study is very good and the statements are reliable because differences of an interdisciplinary concept with psychosomatic stabilization were elaborated on a rather conventional therapy and this therapy is clearly more successful.

#### 5.5.3 Tinnitus specific cognitive and manualized behavioral therapy

Cognitive behavioral therapies are often successfully applied in tinnitus patients but they are not really standardized. Zenner and co-workers presented a cognitive behavioral therapy that is performed specifically for tinnitus in a strictly manualized way. In a controlled multicenter trial, 286 patients with tinnitus persisting for at least 4 months were evaluated [212]. The waiting group (n=120) was the control group. 84% of the treated tinnitus patients improved, but only 22% of the control group. Improvements in the tinnitus questionnaire, the tinnitus loudness and severity were significantly higher in the therapy group than in the control group. In the tinnitus questionnaire, the therapy group improved its score from 27 to 13.5 on average, the control group had no improvement.

The effectiveness of cognitive behavioral therapy was confirmed with sufficient evidence in cases of chronic and decompensated tinnitus. However, the severity is reduced by treatment of the comorbidities such as sleep disorders, anxiety, and depression. The manualized behavioral therapy evaluated here, that focuses especially on tinnitus and thus has tinnitus-specific parts in particular in the psycho-education, is certainly effective as described in the presented trial. But no catamnestic data was collected so that the long-term effect is not proven. Furthermore, control groups consisting of waiting lists are better than no control but they are unspecifically subject to other influences, e.g. the expectation of improvement or the anger and despair about the waiting time.

The problem of manualized psychotherapy is always the missing individuality and thus also specificity, in contrast it can be better evaluated and compared. However, it is astonishing that existing hearing loss was not considered in this study, although it has a high impact on the effect of tinnitus therapy depending on the degree of rehabilitation of the hearing loss.

#### 5.5.4 Is short-term therapy helpful?

The brief intervention for acute tinnitus presented by the group of Göttingen is based on a cognitive behavioral approach: 304 patients were randomly assigned to 4 groups. One group received internet-based instructions for behavioral therapy, another one a book-based (counseling) therapy, the third group underwent group therapy, and the forth one had only information. The tinnitus severity decreased significantly for the internet and group therapy groups compared to the control groups. This is also true for a subsequently performed investigation where group therapy provided the best results [213]. In a prospective study, 93 patients with chronic decompensated tinnitus were evaluated who received multimodal therapy for 5 days. The patients improved significantly but the actual effect size was low. The authors postulate that short-term therapy may be effective but more intensive treatment approaches are more effective and longer-lasting [214].

Even cheaper would be a therapy that is internet-based and thus does not require personnel. In Sweden, such an internet-based therapy was evaluated and compared in a review article with direct, personal cognitive behavioral therapy [215]. 13 studies with a total of 1,053 patients were evaluated; direct cognitive behavioral therapy was often superior or it could not be directly compared. Nonetheless, the authors drew the conclusion that an internet-based cognitive therapy for tinnitus could be an effective option for the future.

Beside the common questionnaires for measuring the therapeutic success also the actual definition of effect sizes is important for the scientific evaluation of therapy results. Even if the results may be good, as shown in an article about short-term therapy, the permanent results are not yet assessed. More intensive therapeutic offers achieve better effect sizes. Also for internet-based therapy, the situation will be similar: it may be effective (short-term) but long-term therapeutic effects can certainly be better achieved via personal intensive single or group therapies.

### 5.6 Neuro-otologic psychosomatic therapies (NPT)

#### Yearly results of inpatient psychosomatic tinnitus therapy based on neuro-otology 

An integrative neuro-otologic and psychosomatic tinnitus therapy as outpatient and even more as inpatient treatment concept is well implemented in practice and turned out to be successful for the treatment of a high number of tinnitus patients. The pre-condition is a long-term treatment in a team where the patient is an actively participating part of the team.

In this context, neuro-otologic diagnostics are imperatively required for every tinnitus patient in order to develop a sound therapy model together with the patient so that treatment can be performed in a reliable therapeutic relationship.

Additionally, a direct therapy of the “symptom of tinnitus” by hearing therapeutic approaches and acoustic stimulation, generally by using hearing aids, is also highly effective so that rather a synergy should be looked for in the sense of an actual psychosomatic therapy.

For therapy [216], the capacities of the human brains are used to achieve plastic changes by the learning processes. For the perception of the background signal of tinnitus, this means an active and permanent habituation. It is promoted by hearing therapeutic units, but it can only be achieved if the signal of tinnitus is not emotionally charged and the patient is psychically sufficiently stabilized. 

In a recent study and evaluation of this therapy, 368 inpatients with complex tinnitus suffering were included. From 2010 to 2015, at the beginning and the end of the treatment, they filled out the tinnitus questionnaire according to Goebel and Hiller (2004) [24] as well as the German version of the Hospitality Anxiety and Depression Score (HADS) [217] for estimation of the anxiety and depression component.

**Figure 1:**
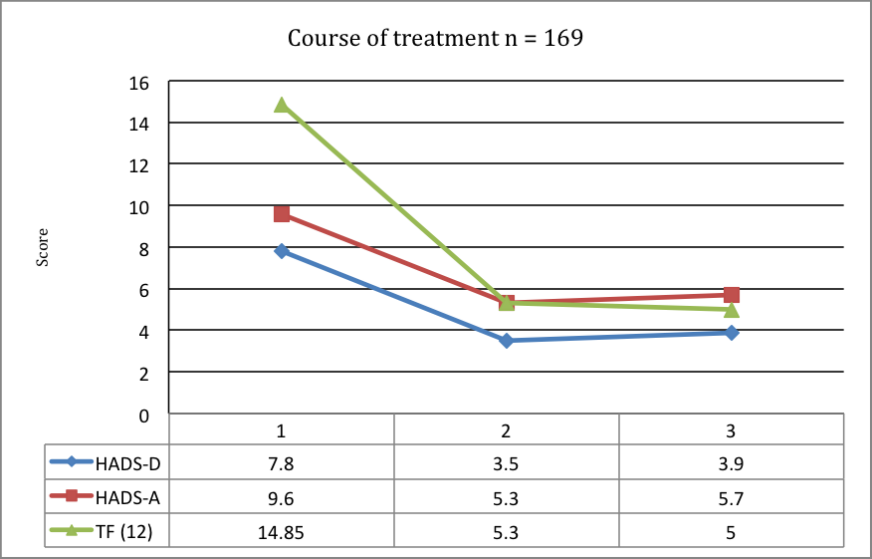
Mini-TF values (green), HADS A (red) and HADS D (blue) at the start of therapy (1), end of therapy (2), and at the time of the follow-up examination (3)

On average, therapy took 38.8 days (SD 13.6), i.e. 5.5 weeks. The mean tinnitus severity at the time of hospitalization was 14.36 (SD 5.8), which corresponds to a moderate severity, at the end it was 5.8 (SD 4.5) corresponding to a mild severity. This difference is highly significant and is equal to an effect size of 2.26 (t(367)=30,627, p<0.001). In the HADS A at the onset of therapy an average of 9.59 (SD 3.7) was measured that could be reduced by treatment to 5.3 (SD 3.6). This is highly significant and corresponds to an highly effective effect size of 1.6 (t(363)=21,568, p<0.001). In the HADS D at the beginning of therapy, an average of 7.98 (SD 4) was measured that could be reduced by treatment to 3.6 (SD 3.1). This is highly significant and corresponds to an highly effective effect size of 1.64 (t(365)=22,183, p<0.001) [216] (Figure 1 (Fig. 1)).

According to all studies and also guidelines, the multimodal, neuro-otologic-psychosomatic tinnitus therapy is certainly a very effective treatment of chronic tinnitus. Especially the multi-disciplinary approach in cooperation of ENT specialists, psychologists, hearing therapists, and acousticians is highly effective. This is clearly confirmed by evaluations of effect sizes.

## 6 Discussion and outlook

Worldwide and especially in Germany, tinnitus therapy positively developed within the last 20 years. In particular, knowledge about central representations and plastic changes in the cortex give way to new therapeutic approaches that focus on the plasticity and the capacity of possible cerebral changes in order to solve reflex-like associated reactions on auditory stimulation and to modify them. This knowledge also considers that tinnitus is a symptom of pathologically altered hearing perception and may be generated by deficits in all parts of the auditory system. Distress and trouble by the tinnitus is not an inevitable result but according to epidemiological data it occurs in less than 25% of the patients. This is due to habituation processes that blind out recurrent, irrelevant stimulations from perception or in contrary reactions of attention and emotional associations accentuating those stimulations and focusing on them. Often such an association of the acoustic troublesome tinnitus with other cerebral regions causes comorbidities of different severity, starting with concentration and sleep disorders up to depressive episodes of different severity, anxiety, or panic reactions. These complex relations and thus most different origins of the development of a disease lead to the fact that a mechanistic understanding of therapy is useless and will not lead to progress in the context of the treatment. Since direct therapy to restitute the hearing capacity is not available, especially when it is the primary result of deficient hair cells of the inner ear, or probably cannot be achieved even after long-term therapy, only the modulation of the central connections and compensation of deficits of hearing processing remains a therapeutic option. However, only by pharmacological interventions, irradiations, electric stimulations, or even maximally invasive procedures such as surgeries, this will never go beyond the effect of placebo because plastic changes in the brain can only take place by learning processes and simultaneous emotional decoupling.

So it is not surprising that according to a review article, among nearly all studies on tinnitus treatment of the last years and decades only those procedures achieve an evidence level that take those complex correlations into account. This is currently only true for cognitive therapies, generally behavioral therapies that are either manualized or even better individually conceived and influence the concrete hearing perception of the patient, co-treat comorbidities, and thus allow habituation of the disturbing signal of tinnitus (or the increased hypersensitivity regarding noise – hyerpacousis). A precondition is a stable relationship between physician and patient and experienced therapists with sound knowledge of the correlations of auditive processing.

Even if the classic evidence criteria are not high for those procedures, they meet the requirements not only of so-called external evidence, confirmed by reliable studies and data, but also correlate concrete experience and needs of physicians and patients. Thus they achieve real evidence. Especially those procedures can only achieve a limited (external) evidence level in studies and meta-analyses because they are nearly never monotherapies and can only be difficultly controlled by placebo.

This article discusses nearly all available therapeutic procedures – indeed, only for very few therapeutic options reliable data and profound studies with methodically excellent planning and control are found. In the context of pharmaceutical studies, those criteria generally have to be met in order to fulfil the strict conditions for admission. But then they confirm always the ineffectiveness or same effect as placebo.

In contrast, nearly all studies on instrument-based, stimulating or regulative interventions are methodically insufficient, they do not meet evidence criteria. This is also true for reliably supportive measures such as hearing aids or cochlear implantations that cannot be evaluated properly, already because they are included in more complex therapeutic options and cannot be controlled by placebo. Here, the external evidence is replaced by clinical experience and continuous integration of the patient in therapy controls.

The same applies to hearing and music therapies. Placebo controls are merely not possible and moreover they are not reasonable. Furthermore they are always related to stabilizing, counseling therapies and relaxation procedures. At the same time they effectively support habituation of the tinnitus.

From the patient’s point of view, the problem of those therapeutic approaches is that they require active cooperation without being able to completely “switch off” the tinnitus. Additionally, they include psychic factors which patients often do not want to admit. So there will always be numerous patients who insist on causal therapies and are ready to take on physical and economical efforts but who finally become more and more frustrated. Since the medical market responds to those requirements, there will always be therapeutic promises arising in fashion waves. Even studies will repeat and gain new generations of scientists or regions of the world (see the example of soft laser therapy) – and again no reliable data will result.

Evidence-based medicine is the “gold standard” and in general evidence may be claimed but it must be critically questioned and especially include clinical experience and the patients’ needs. It must not only refer to studies, in particular if those studies investigate only certain aspects for evidence and neglect other parts of a multimodal therapy concepts – which happens more and more often (e.g. music therapy). An even bigger problem is the inflationary quantity of publications in newly appearing journals and online publications that are not based on proper peer-reviews and controls. Nearly every second day, the author of this contribution receives invitations from online journals to urgently submit papers, the editors promise rapid procedures. Half of those invitations comes from journals that are related to ENT in some respect.

At the same time, studies are rejected by a reviewer of a journal and nonetheless they appear unchanged in another journal that is less strict in its review process (e.g. acoustic stimulation with sounds or combination therapies).

Generally, it must be understood for therapy of chronic diseases (e.g. especially the treatment of chronic pains) and thus also the chronic tinnitus that they are often psychosomatically overlaid and so more or less clearly psychotherapeutic factors have to be included in the therapy, in the form of coaching, support, stabilization, or direct psychotherapeutic intervention.

In summary of this article, it can be stated that the evidence situation for the whole topic of tinnitus therapy is not very good. Evidence gaps are most obvious for nearly all procedures promising direct, causal treatment and for all pharmacological procedures as well as interventions that intend influencing directly the primary or secondary auditory cortex or other brain regions.

Nonetheless, there are psychotherapeutic cognitive procedures based on scientific evidence that can be recommended for the treatment of chronic tinnitus, especially when ENT-specific competence and knowledge on hearing processing and improvement are included.

## Notes

### Competing interests

The author declares that he has no competing interests.
